# Acupuncture for migraine: a literature review of neuroimaging studies

**DOI:** 10.3389/fneur.2025.1601554

**Published:** 2025-06-25

**Authors:** Dan Tong, Rui Yao, Xinyue Zhang, Siyuan Tao, Nuo Chen, Wanxia Wu, Sai Wu, Jun Zhou, Yulan Ren, Fanrong Liang, Litao Pan, Zhengjie Li

**Affiliations:** ^1^Acupuncture and Tuina School, Chengdu University of Traditional Chinese Medicine, Chengdu, China; ^2^Department of Acupuncture and Massage, The First Affiliated Hospital of Shenzhen University, Shenzhen Second People’s Hospital, Shenzhen, China; ^3^Acupuncture & Brain Research Center, Chengdu University of Traditional Chinese Medicine, Chengdu, China; ^4^Key Laboratory of Acupuncture for Senile Disease (Chengdu University of TCM), Ministry of Education, Chengdu, China

**Keywords:** migraine, neuroimaging mechanisms, acupuncture, functional magnetic resonance imaging, literature review

## Abstract

**Background:**

Acupuncture is effective for migraine treatment with minimal side effects. Neuroimaging techniques have been extensively used to explore the central mechanisms of acupuncture for migraine. This review aims to summarize neuroimaging study of acupuncture for migraine, providing a valuable reference and foundation for future clinical and mechanistic research.

**Methods:**

From database inception to November 19, 2024, we conducted a comprehensive search of four English-language and four Chinese-language databases. All published clinical controlled trials utilizing neuroimaging methods were included after rigorous screening. This review summarizes the immediate and preventive effects of acupuncture in migraine treatment, as well as the possible neural mechanisms underlying its efficacy in alleviating pain and non-pain symptoms.

**Results:**

A total of 833 papers were identified, and 40 met the inclusion criteria after screening. Among them, 8 studies focused on the immediate effects of acupuncture, while 32 investigated its preventive effects. General, methodological, and neuroimaging data were extracted and summarized. These studies utilized various neuroimaging techniques, including functional magnetic resonance imaging (fMRI), diffusion tensor imaging (DTI), fluorodeoxyglucose positron emission tomography/computed tomography (FDG-PET/CT), transcranial Doppler sonography (TCD), and magnetic resonance spectroscopy (MRS). The immediate effects of acupuncture treatment for migraines primarily involve brain regions such as the middle frontal gyrus (MFG), precuneus, and postcentral gyrus, most of which belong to the default mode network (DMN), central executive network (CEN), and salience network (SN). In terms of preventive effects, the key regions involved are the precuneus, anterior cingulate cortex (ACC), MFG, most of which involved in the DMN, SN, CEN, and descending pain modulatory system (DPMS).

**Conclusion:**

This systematic review reveals that the brain regions involved in acupuncture treatment for migraine headache include the DMN, SN, CEN, and DPMS, which are related to pain sensation, emotion and modulation. Future research could prioritize meticulously designed, high-quality, multimodal neuroimaging studies to further elucidate the neuroimaging evidence for acupuncture in migraine treatments from different aspects.

## Introduction

Migraine is a disabling neurological disorder that imposes a significant burden on both individuals and society (1, 2). It is primarily characterized by moderate to severe unilateral throbbing and pulsating headaches (3), and may also be accompanied by non-headache symptoms such as nausea, vomiting, photophobia, and phonophobia (4). Although the mechanisms triggering migraine attacks are not fully understood, migraine is believed to involve the activation and sensitization of the trigeminovascular system, brainstem, diencephalon, and cortical regions (5).

Currently, migraine pharmacotherapy is divided into acute treatment and preventive treatment (6, 7). Medications commonly used for the acute treatment of migraines include nonsteroidal anti-inflammatory drugs (NSAIDs) and triptans (8). However, NSAIDs are not specifically developed for migraine treatment and are associated with multiple side effects, such as gastrointestinal toxicity and cardiovascular risks (6, 9). In addition, more than 20% of patients have cardiovascular contraindications to triptans (10). Medications typically used for migraine prevention include antihypertensives, anticonvulsants, and antidepressants, but their use may be limited due to potential contraindications, drug interactions, limited efficacy, or a high risk of adverse effects (7, 11). In contrast, non-pharmacological treatments (such as acupuncture, non-invasive or invasive neuromodulation, mind–body treatments, and behavioral treatments) are gaining increasing attention due to their lack of side effects and sustainability (12, 13). Acupuncture, as a traditional Chinese medical therapy, has demonstrated significant advantages in the treatment of migraines (14–16). Previous studies have shown that acupuncture can effectively reduce the frequency and severity of migraine attacks by modulating neurovascular function, alleviating inflammatory responses, and balancing neurotransmitter levels (17). What’s more, acupuncture has virtually few side effects and has demonstrated long-term benefits in some patients, enabling patients to achieve preventative symptom relief without relying on medications. The safety and efficacy of acupuncture make it an important treatment option for migraine patients. However, the mechanisms by which acupuncture relieves migraines remain largely unknown.

Recent years, neuroimaging technology has advanced rapidly, offering a powerful tool for the study of migraine non-invasively. For example, functional magnetic resonance imaging (fMRI) can capture functional changes in the brains of migraine patients, enhancing our understanding of the pathophysiology of migraine (18). Transcranial Doppler sonography (TCD) can non-invasively and cost-effectively examine the blood flow velocity of the basilar artery and the characteristics of cerebral hemodynamics (19). Diffusion tensor imaging (DTI) is an advanced MRI technique used to provide qualitative and quantitative information about white matter microstructure (20). Magnetic resonance spectroscopy (MRS) is used to assess the levels of specific neurotransmitters and study changes in brain metabolites (21). Fluorodeoxyglucose positron emission tomography/computed tomography (FDG-PET/CT) primarily displays glucose metabolism in different brain regions of migraine patients (22). Notably, a large number of researchers have used these techniques to conduct fruitful studies on the central mechanisms of acupuncture for migraine. However, there is a scarcity of systematic reviews summarizing these studies, and existing reviews have not comprehensively analyzed the characteristics of neuroimaging study designs (23, 24).

Consequently, this systematic review study aims to address two core questions: (1) the neuroimaging mechanisms underlying acupuncture treatment for migraines, and (2) providing a reference and foundation for future clinical and mechanistic research on acupuncture for migraine treatment. We hope that a comprehensive summary of the existing studies will provide a more scientific theoretical basis for the treatment of migraine and promote the further development of acupuncture in clinical applications.

## Methods

### Search strategies

We searched for articles published from the establishment of the databases to November 19, 2024 in the electronic English databases such as PubMed, Embase, Cochrane Library, Web of Science, as well as in the Chinese databases including China National Knowledge Infrastructure (CNKI), VIP Database (VIP), Wanfang Database (WF) and China Biomedical Literature Database (CBM). The keywords for retrieval included “migraine,” “acupuncture,” “neuroimaging,” etc. See Supplementary material for the details of all search strategies.

### Inclusion criteria

The articles we included were required to meet the following criteria: (1) Study types: All published controlled clinical studies were included. Only articles in English and Chinese were considered. (2) Participants: Patients who met the diagnosis criteria for migraine set by the International Headache Society (IHS). (3) Intervention measures: Manual acupuncture (MA) or electroacupuncture (EA). (4) Brain imaging studies: Technologies such as magnetic resonance imaging (including fMRI and structural MRI), PET, DTI, magnetoencephalography (MEG), electroencephalogram and functional near-infrared spectroscopy imaging were involved.

### Exclusion criteria

We excluded articles which were written in other languages, animal experiments or irrelevant to acupuncture, migraine or neuroimaging. We also excluded articles that did not conform to the required study types, such as meta-analyses, reviews, or case reports.

### Study selection

**Figure 1:**
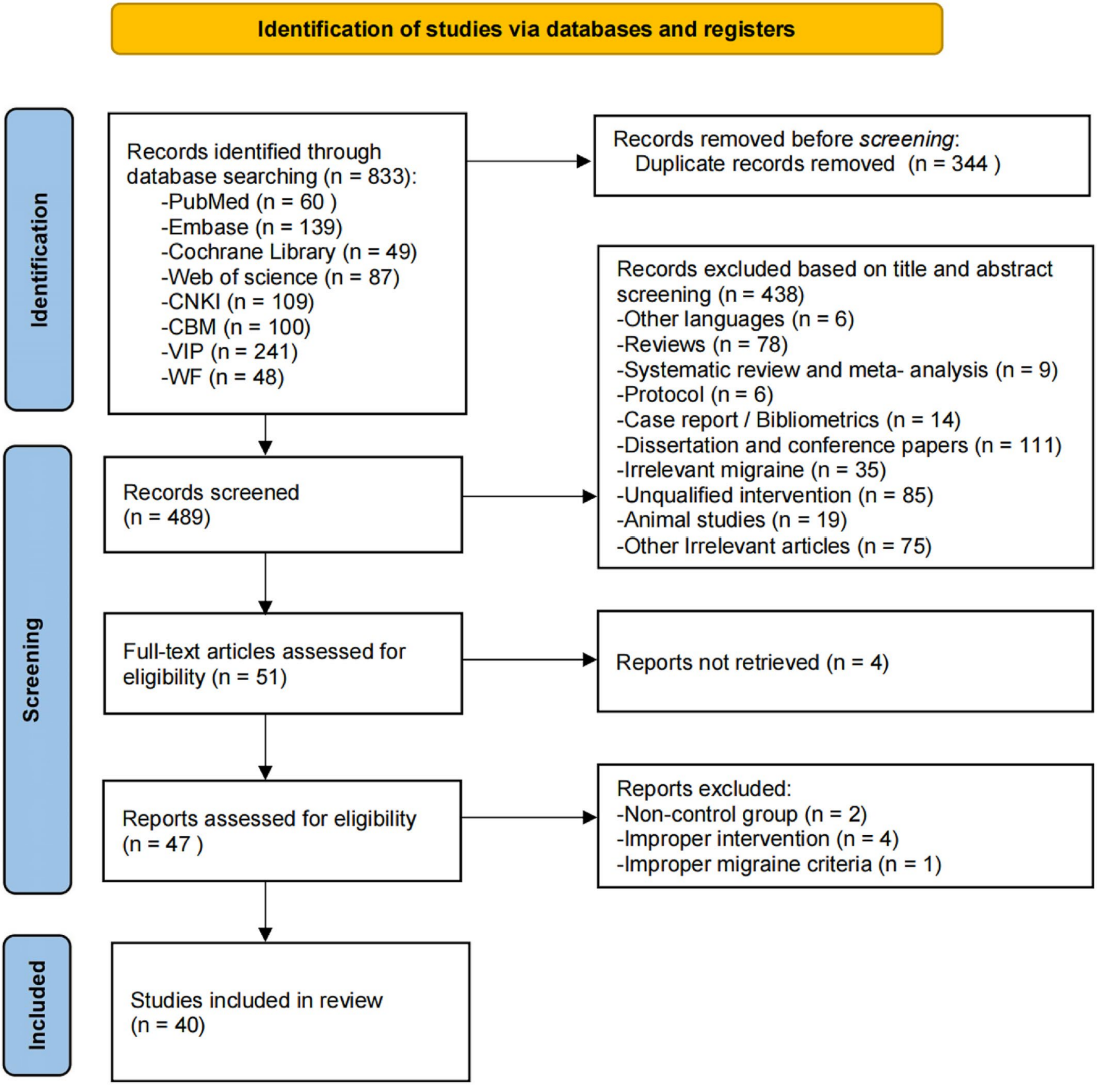
The flow diagram of literature screening.

Through the search strategies, a total of 833 articles were retrieved, with 60 from PubMed, 139 from Embase, 49 from Cochrane Library, 87 from Web of Science, 109 from CNKI, 100 from CBM, 241 from VIP and 48 from WF. The articles retrieved through the search strategy were imported into EndNote 21 for screening and duplicate removal. After excluding 344 duplicate articles, the remaining 489 articles were screened based on their titles and abstracts. Among these, 438 articles were further excluded for the following reasons: 6 were other languages, 78 were review or synthesis articles, 9 were systematic reviews or meta-analyses, 6 were research protocols, 14 were case reports or bibliometric studies, 111 were dissertations or theses, 35 were irrelevant to migraine, 85 were unrelated to acupuncture, 19 were animal studies, and 75 were irrelevant to the study’s objectives. The full texts of the remaining 51 articles were thoroughly analyzed. Subsequently, 4 articles were excluded due to incomplete information, 2 were excluded for lacking a control group, 4 were excluded for incompatible interventions, and 1 was excluded for not meeting the migraine diagnosis inclusion criteria. Ultimately, 40 research articles were identified for inclusion in this study. See Figure 1 for the study inclusion flowchart.

### Data extraction

Authors DT and RY independently evaluated the retrieved articles and extracted data. The data were compiled into a standardized data extraction form in an Excel spreadsheet, listing aspects like the year, migraine subtypes, sample size, acupuncture parameters, neuroimaging techniques, clinical research results, etc. Any discrepancies were resolved through discussions among the authors. If no consensus could be reached, the authors’ team would be responsible for facilitating an agreement.

## Results

### Study characteristics of the neuroimaging studies

**Figure 2:**
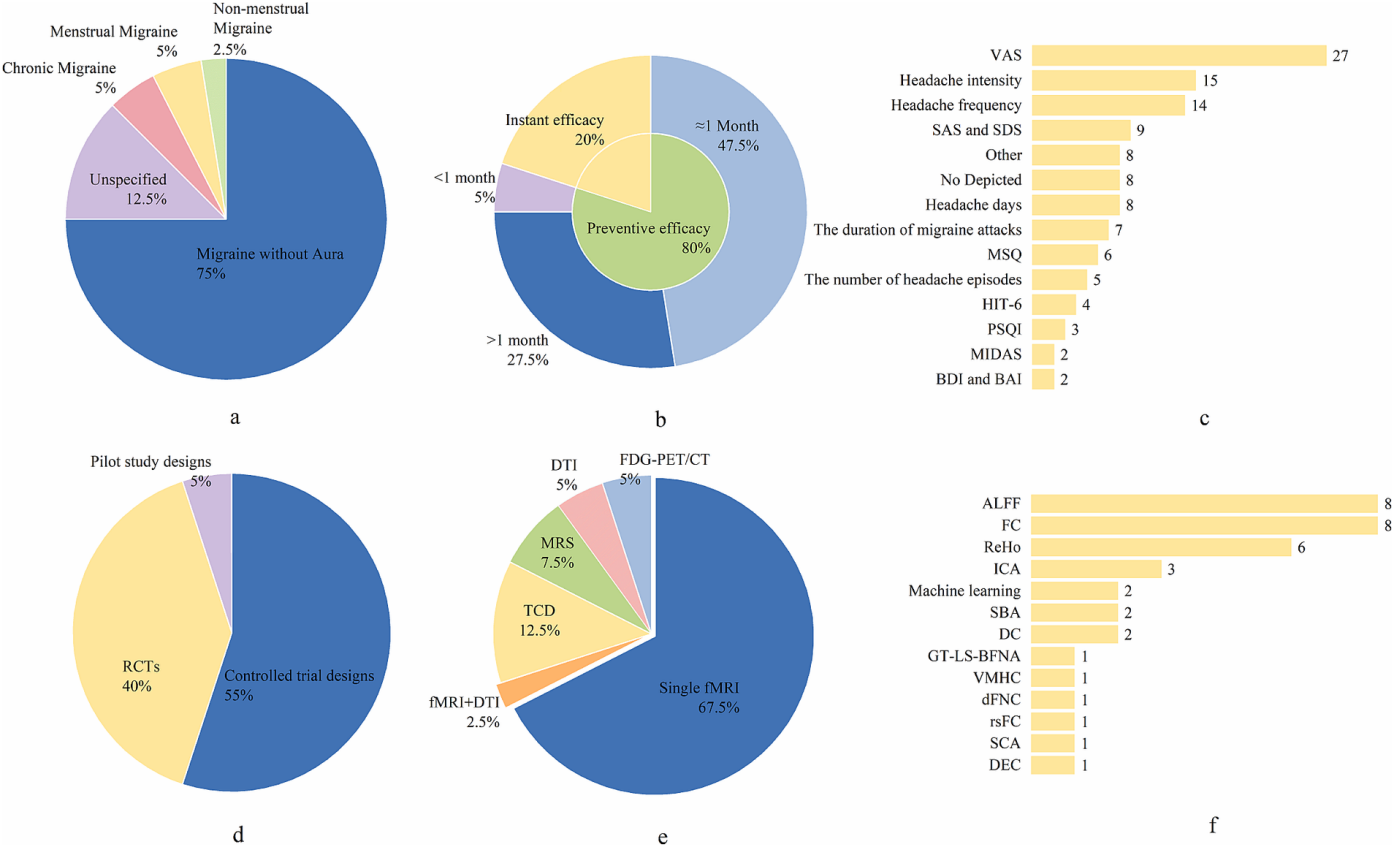
The research design of the included studies. **(a)** The proportion of various types of migraine. **(b)** The proportion of the number of acupuncture—treatment sessions. **(c)** The proportion of the variables for clinical efficacy assessment of migraine. **(d)** The proportion of different trial design types. **(e)** The proportion of neuroimaging techniques employed. **(f)** The proportion of the data—analysis modalities for neuroimaging. VAS, Visual Analogue Scale; SAS, Self-Rating Anxiety Scale; SDS, Self-Rating Depression Scale; MSQ, Migraine-Specific Quality of Life Questionnaire; HIT-6, Headache Impact Test-6; PSQI, Pittsburgh Sleep Quality Index; MIDAS, Migraine Disability Assessment Questionnaire; BDI, Beck Depression Inventory; BAI, Beck Anxiety Inventory; RCTs, randomized controlled trials; fMRI, functional magnetic resonance imaging; DTI, diffusion tensor imaging; FDG-PET/CT, Fluorodeoxyglucose Positron Emission Tomography/Computed Tomography; MRS, magnetic resonance spectroscopy; TCD, transcranial Doppler; ALFF, amplitude of low-frequency fluctuations; FC, functional connectivity; ReHo, regional homogeneity; ICA, independent component analysis; SBA, seed-based analysis; DC, dynamic causal modeling; GT-LS-BFNA, graph theory-based large-scale brain functional network analysis; VMHC, voxel-mirrored homotopic connectivity; dFNC, dynamic functional network connectivity; rsFC, resting-state functional connectivity; SCA, spectral clustering analysis; DEC, dynamic effective connectivity.

The database search identified 833 studies. After excluding duplicates, reviews, meta-analyses, animal studies, research protocols, and other irrelevant articles, 40 papers met the inclusion criteria for further evaluation. For each included study, we systematically extracted the following data: authors, publication year, country, study design, type of migraine, diagnostic criteria, sample size, age range, gender (male/female), acupuncture intervention parameters (e.g., needle retention time, frequency, and acupoints selected), neuroimaging technology, and clinical outcomes. Comprehensive details are summarized in Table 1 and Figure 2.

**Table 1:** Details of the study design of acupuncture for the treatment of migraine.

Author, year, country	Study design	Type of migraine	Diagnostic criteria	N (A/B/C)	Age (Year)	Gender (M/F)	Treatment courses	(A)	(B)	(C)	Acupoints	Acupuncture parameters	Neuroimaging technology	Clinical outcomes
								Intervention	Control Group I	Control Group II				
Yang et al. (2012), China (22)	RCT	Acute MWoA	IHS (version not mentioned)	30 (10/10/10)	32.87 ± 8.71	12/18	30 min	Traditional acupuncture	Control acupuncture	Migraine	A: SJ5, GB34, GB20	EA: Deqi, electrodes on auxiliary needles punctured 2 mm lateral to the points and 2 mm in depth, 100 Hz, 0.1–1.0 mA, 30 min, once	FDG-PET/CT	VAS
											B: ST8, LI6, ST36			
											C: No treatment			
Yang et al. (2014), China (27)	RCT	Acute MWoA	IHS (version not mentioned)	30 (10/10/10)	33.28 ± 8.03	12/18	30 min	Acupuncture	SA	Migraine	A: TE19, SJ8, GB33	EA: Deqi, electrodes on auxiliary needles punctured 2 mm lateral to the points and 2 mm in depth, 100 Hz, 0.1–1.0 mA, 30 min, once	FDG-PET/CT	VAS
											B: Non-acupoints			
											C: No treatment			
Liu et al. (2016), China (30)	Controlled trial	MWoA	ICHD-2	28 (15/13)	A:28.7 ± 6.1	A:3/12	9 min 10 s	Acupuncture	HC	/	Bilateral: GB41	0.5–0.8 inch, the needle was twisted for 1 min after 10s, twisting 180°, 100 times/min, and then left for 8 min	fMRI (FC)	/
					B: Match with A	B: Match with A								
Ning et al. (2017), China (28)	Controlled trial	MWoA	IHS (version not mentioned)	32 (16/16)	A:28.3 ± 6.0	A:3/13	60s	Acupuncture	HC	/	Bilateral: GB41	2–3 cm, rotating the needle clockwise and counterclockwise at 1 Hz, reinforcing and reducing manipulation for 60s	rs-fMRI (ALFF)	/
					B:27.1 ± 4.8	B:3/13								
Han et al. (2017), China (31)	Controlled trial	MWoA	ICHD-2	20 (10/10)	A:31.7	A:2/8	10 min 10 s	Acupuncture	HC	/	Bilateral: GB41	0.5–0.8 inch, 10 min and 10 s	fMRI (ReHo)	/
					B: Match with A	B: Match with A								
Ning et al. (2020), China (29)	Controlled trial	MWoA	ICHD-2	37 (19/18)	A:28.23 ± 6.16	A:3/16	9 min	Acupuncture	HC	/	Bilateral: GB41	The needle was twisted for 1 min in the flat tonic and flat diarrhea method, and then left for 8 min	fMRI (ALFF)	/
					B:27.16 ± 5.23	B:4/14								
Wei et al. (2022), China (32)	Pilot study	MWoA	ICHD-3 beta	60 (30/30)	A:33.64 ± 6.87	A:5/25	8 min	Acupuncture	HC	/	GB8	EA:2 Hz, 1 mA, 15° angle, 0.5 inches, 8 min, once	rs-fMRI (FC)	VAS, HIT-6, HRSD-17, HRSA-14
					B:30.93 ± 5.95	B:5/25								
Luo et al. (2022), China (33)	Controlled trial	MWoA	ICHD-3	40 (20/20)	A:35.48 ± 7.90	A:4/16	10 min	Acupuncture	HC	/	Bilateral: GB8	EA: Deqi, continuous wave, 2 Hz, 1–2 mA, 15° angle, 0.5 inches, stimulated state 30 s, non-stimulated state 2 min, 4 times	rs-fMRI (FC)/ts-fMRI	/
					B:29.93 ± 8.30	B:4/16								
Bäcker et al. (2004), Germany (34)	Pilot study	Migraine	IHS (version not mentioned)	20 (10/10)	A:29–48	/	2 weeks	Acupuncture	HC	/	Bilateral: GB41, LR3, SJ5, EX-HN5, SJ23, DU20, GB20	10 sessions, twice a week for the first 4 weeks and once a week for the following 2 weeks, 30 min	fTCD	Headache frequency and intensity
					B: Match with A									
Zhao et al. (2014), China (43)	RCT	MWoA	ICHD-2	40 (20/20)	A:32.9 ± 10.99	A:6/14	8 weeks	Active acupuncture	Inactive acupuncture	/	A: Bilateral: SJ5, GB20, GB34, and GB40	2.5–3.5 cm, rotation (90° < amplitude<180°) at a frequency of 1–2 Hz, Deqi, 4 sessions per week, 30 min	rs-fMRI (ReHo)	The intensity of headache (VAS), duration and severity of headache, HIT-6
					B:37.25 ± 9.68	B:8/12					B: Bilateral: SJ22, PC7, GB37, and SP3			
Li et al. (2015), China (72)	Controlled trial	MWoA	IHS (version not mentioned)	24 (12/12)	A:28.1 ± 6.8	A:2/10	4 weeks	Acupuncture	HC	/	Bilateral: SJ23, GB8, GB20, EX-HN5, LI4, LR3, SJ5, GB34, and GB41	1.5–2.5 cm, Deqi, 5 times per week, 30 min	fMRI (FC)/DTI	VAS, duration and frequency of migraine attacks
					B:29.8 ± 7.2	B:2/10								
Zhang et al. (2016), China (73)	Controlled trial	MWoA	ICHD-2	24 (12/12)	A:28.1 ± 6.8	A:2/10	4 weeks	Acupuncture	HC	/	Bilateral: SJ23, GB8, GB20, EX-HN5, LI4, LR3, SJ5, GB34, and GB41	1.5–2.5 cm, Deqi, 5 times per week, 30 min	fMRI (ICA)	The intensity of headache (VAS), duration and frequency of migraine attacks, PSQI
					B:29.8 ± 7.2	B:2/10								
Li et al. (2016), China (48)	RCT	MWoA	ICHD-2	104 (62/42)	A:21.29 (20.85; 21.73)	A:14/48	4 weeks	A1: VA1, VA2, VA3 A2: SA A3: WT	HC	/	Bilateral: VA1: GB34, GB40, SJ5 VA2: GB33, GB42, SJ8 VA3: ST36, ST42, L16 SA: NAP1, NAP2, NAP3	5–15 mm, Deqi, once per day for five weekdays, 30 min	fMRI (SBA)	The intensity of headache (VAS), frequency of migraine attacks, SAS, SDS
					B:21.21 (20.93; 21.49)	B:8/34								
Li et al. (2017), China (47)	RCT	MWoA	ICHD-2	104 (62/42)	A:21.29 (20.85; 21.73)	A:14/48	4 weeks	A1: VA1, VA2, VA3 A2: SA A3: WT	HC	/	Bilateral: VA1: GB34, GB40, SJ5 VA2: GB33, GB42, SJ8 VA3: ST36, ST42, L16 SA: NAP1, NAP2, NAP3	5–15 mm, Deqi, once per day for five weekdays, 30 min	fMRI (ALFF)	The intensity of headache (VAS), frequency of migraine attacks, SAS, SDS
					B:21.21 (20.93; 21.49)	B:8/34								
Li et al. (2017), China (49)	RCT	MWoA	ICHD-2	118 (72/46)	A:21.30 (20.89; 21.73)	A:15/57	4 weeks	A1: VA1, VA2, VA3 A2: SA A3: WT	HC	/	Bilateral: VA1: GB34, GB40, SJ5 VA2: GB33, GB42, SJ8 VA3: ST36, ST42, L16 SA: NAP1, NAP2, NAP3	5–15 mm, Deqi, once per day for five weekdays, 30 min	fMRI (ICA, SBA)	The intensity of headache (VAS), frequency of migraine attacks, SAS, SDS
					B:21.24 (20.98; 21.50)	B:10/36								
Gu et al. (2018), China (39)	Controlled trial	MWoA	ICHD-2	29 (15/14)	A:34.8 ± 10.6	A:5/10	5 weeks	Acupuncture	HC	/	DU20; bilateral: GB20, LR2	EA: dilatational wave, 1.5–2.5 cm, Deqi, 5 sessions, once per week, 30 min	MRS	Headache intensity (VAS), frequency of headache attacks, duration of headache attacks
					B:22.4 ± 1.1	B:4/10								
Zou et al. (2019), China (53)	Controlled trial	CM	ICHD-3 beta	32 (14/18)	A:42.91 ± 10.18	A:5/9	3 months	Acupuncture	HC	/	Bilateral: SJ5, GB20, GB8, ST8	5–15 mm, Deqi, 3 sessions per week, 30 min	fMRI (ICA, SCA)	VAS, headache attacks, headache days, acute headache medications, immediate VAS
					B:38.59 ± 7.96	B:9/9								
Tu et al. (2020), China (74)	RCT	MWoA	ICHD-2	116 (70/46)	A:21.5 ± 2.0	A:15/55	4 weeks	A1: RA1, RA2, RA3 A2: SA A3: WT	HC	/	Bilateral: RA1: GB34, GB40, SJ5 RA2: GB33, GB42, SJ8 RA3: ST36, ST42, L16 SA: NAP1, NAP2, NAP3	5–15 mm, Deqi, once per day for five weekdays, 30 min	fMRI (FC), machine learning analyses	/
					B:21.2 ± 0.9	B:9/37								
Yin et al. (2020), China (75)	Controlled trial	MWoA	IHS (version not mentioned)	80 (40/40)	A:21.7 ± 2.15	A:10/30	4 weeks	Acupuncture	HC	/	Bilateral: Prescription 1: GB34, GB40, SJ5 Prescription 2: GB33, GB42, SJ8 Prescription 3: ST36, ST42, L16	Deqi, once per day for five weekdays, 30 min	fMRI (zALFF), machine learning analyses	The frequency of migraine attack, headache intensity (VAS), MMDs
					B:21.1 ± 2.01	B:10/30								
Tian et al. (2021), China (76)	Controlled trial	MWoA	IHS (version not mentioned)	96 (48/48)	A:21.17 ± 0.93	A:11/37	4 weeks	Acupuncture	HC	/	Bilateral: Prescription 1: GB34, GB40, SJ5; Prescription 2: GB33, GB42, SJ8; Prescription 3: ST36, ST42, L16	5–15 mm, Deqi, 30 min, 5 sessions per week	rsfMRI (FC)	Headache duration, headache intensity (VAS), headache frequency, SAS, SDS
					B:21.29 ± 1.89	B:11/37								
Liu et al. (2021), China (45)	Controlled trial	MWoA	ICHD-3	52 (37/15)	A:37.97 (9.82)	A:6/31	6 weeks	Acupuncture	HC	/	DU20, EX-HN5, bilateral GB20, GB8, GB5, GB15, LI4, and LR3 EA:bilateral GB20, GB8	EA: Deqi, bilaterally at GB20 and GB8, 2 Hz, 0.1–1.0 mA, twice a week, 20 min	rsfMRI (ReHo)	Migraine days, VAS, SAS, SDS, MSQ
					B:34.88 (6.66)	B:2/13								
Zhang et al. (2021), China (46)	RCT	Menstrual migraine without aura (MMoA)	ICHD-3 beta	44 (24/20)	A:33.04 ± 6.43	All female	3 months	TA	SA	/	A: Bilateral: GB20, GB8, PC6, SP6, LR3	0.5–1.5 cm, twisted and rotated (90° < amplitude <180°), 1–2 Hz, Deqi, 9 ± 2 times a month, 30 min	rsfMRI (ALFF, ReHo)	SAS, SDS, attack frequency, VAS, intensity of the migraine
					B:35.30 ± 9.43						B: Non-acupuncture points			
Liu et al. (2022), China (52)	RCT	MWoA	ICHD-3 beta	40 (20/20)	A:38.60 (12.27)	A:3/17	4 weeks	RA	SA	/	A: Bilateral: GB20, EX-HN5, LI4, GV16, GV20	10–15 mm, Deqi, twist 90–180°, lift and thrust 3–5 mm, 10s every 10 min to maintain the Deqi sensation, 3 sessions per week, 30 min	fMRI (Seed-to-voxel rsFC)	MMDs, VAS, HIT-6, MSQ, BDI-II, BAI, PSQI, and MoCA
					B:36.10 (9.61)	B:1/19					B: Non-acupuncture points			
Chen et al. (2022), China (77)	Controlled trial	MWoA	ICHD-3	73 (40/33)	A:38.02 ± 9.79	A:6/34	6 weeks	Acupuncture	HC	/	DU20, EX-HN5, bilateral GB20, GB8, GB5, GB15, LI4, and LR3 EA:bilateral GB20, GB8	EA: Deqi, bilaterally at GB20 and GB8, 2 Hz, 0.1 to 1.0 mA, 12 sessions, twice a week, 20 min	fMRI (dALFF, DEC)	The frequency of migraine attacks, VAS, SAS, SDS, and MSQ
					B:33.26 ± 5.76	B:7/26								
Wallasch et al. (2012), Germany (35)	RCT	Migraine	ICHD-2	35 (18/17)	A:37.2 (9.6)	A:2/16	8 weeks	VA	Placebo acupuncture	/	A: Bilateral: LI4, ST36, SJ5, GB41, SI3, UB62, DU20, GB20, EX-HN5, SJ23, LR3, KI3	Deqi, one session each week, 30 min	fTCD	Headache frequency and intensity
					B:39.3 (11.7)	B:2/15					B: Areas of the skin that were outside a classically described acupuncture point (minimum 1–2 cm beside)			
Li et al. (2023), China (42)	RCT	Migraine	ICHD-3	38 (28/10)	A1:37.4 (11.0) A2:33.0 (7.5) A3:35.7 (6.6)	A:12/16	11 days	A1: Acupuncture A2: SA A3: Blank control	HC	/	A1: GB20, LR3, EX-HN5, GV20, EX-HN1 A2: NAP A3: No treatment	Deqi, lift and 0.3–0.5 cm, 60–90 times/min, twist 90–180°, two courses of treatment, each of which lasted 5 days, with an interval of 1 day between the two courses, 30 min	fMRI (ALFF, ReHo)	VAS, PSQI, MSQ, GAD-7, attack frequency
					B: 30.4 (7.2)	B:3/7								
Quan et al. (2024), China (78)	Controlled trial	MWoA	ICHD-3	243 (143/100)	A:28.20 ± 0.82	A:36/107	8 weeks	Acupuncture	HC	/	Two obligatory points (GB20 and GB8) were used, and the other two points (SJ5, GB34, BL60, SI3, LI4, ST44, LR3, and GB40) were selected according to the syndrome differentiation of meridians in the headache region	EA:2/100 Hz (alternating every 3 s), 0.1–1.0 mA, 24 sessions, three sessions per week, 30 min	fMRI (dFNC)	Headache frequency, NRS, SAS, SDS, MSQ
					B:27.76 ± 0.13	B:26/74								
Yang et al. (2024), China (51)	Controlled trial	Non-menstrual MWoA	ICHD-3	100 (50/50)	A:28.36 ± 3.61	All female	4 weeks	Acupuncture	HC	/	DU20, GB8, GB5, ST8	EA: Deqi, applied at DU20 and GB8 on the affected side, 2 Hz, 0.1 to 1.0 mA, 2 sessions per week, 20 min	BOLD fMRI (ALFF, DC)	The MIDAS and SF-MPQ scores
					B:27.60 ± 3.02									
Qin et al. (2019), China (44)	RCT	MWoA	ICHD-3 beta	38 (19/19)	A:30.5 ± 6.8	A:3/16	4 weeks	Acupuncture	SA	/	A: Bilateral headache-acupoint	25–40 mm deep, to achieve discharge sensation, numbness and distension of the sensation immediately out of the needle, 45°angle, once a day	fMRI (ReHo)	VAS
					B:31.7 ± 7.2	B:5/14					B: The non-acupoint area on the dorsal side of the foot			
Liu et al. (2022), China (79)	Controlled trial	MWoA	ICHD-3	50 (34/16)	A:40 ± 10	A:7/27	6 weeks	Acupuncture	HC	/	GV20, GB5, GB8, EX-HN5, GB20, GB15, LI4, LR3	EA: The acupuncture area has a sense of acid swelling and heaviness, 2 Hz, 0.1–1.0 mA, 2 times per week, 20 min	rsfMRI (FC)	Headache days, VAS, the total score of headache symptoms, SAS, SDS, MSQ
					B:35 ± 7	B:2/14								
Fu et al. (2024), China (37)	Controlled trial	MWoA	ICHD-3	25 (15/10)	A:37.53 ± 7.15	A:4/11	4 weeks	Acupuncture	HC	/	Affected side: SJ23, GB8, EX-HN5, GB20, bilateral: LI4, LR3, GB41, GB34, SJ5, LI11, LI10	Deqi, 3 times per week, 30 min	MRS	Days of headache episodes, number of episodes, and severity of headache (VAS)
					B:36.80 ± 6.51	B:2/8								
Zheng et al. (2013), China (26)	RCT	MWoA	ICHD-2	42 (22/20)	/	/	4 weeks	Shaoyang points	Non-acupuncture points	/	A: Unilateral: GB20, SJ5, GB34, GB40, alternating left and right sides	EA: Insert an auxiliary needle 2 mm shallow at the proximal end of the acupuncture site and connect the electrode	TCD	Headache days, headache counts, VAS
											B: Non-acupuncture points			
Liang et al. (2016), China (38)	RCT	CM	ICHD-2	60 (30/30)	A: Average age 46.5	A:11/19	4 weeks	Shaoyang points	Non-acupuncture points	/	A: Bilateral: GB34, SJ20, GB40, SJ5	Twist 90–180°, 60–90 times/min, 5 times for a course of treatment, 1 time a day, every 5 times rest 2 d, 30 min	H-MRS	VAS
					B: Average age 44.5	B:10/20					B: Non-acupuncture points next to the meridian points taken by group A			
Chen et al. (2009), China (36)	RCT	Migraine	ICHD-2	59 (30/29)	A:28.07 ± 0.83	A:8/22	4 weeks	Shaoyang points	Non-acupuncture points	/	A: SJ20, SJ5, GB34, GB40	Twist 90–180°, 60–90 times/min, once a day, 5 times for 1 course of treatment, rest 2d between courses	TCD	/
					B:26.86 ± 0.85	B:12/17					B: Non-acupuncture points			
Lin et al. (2013), China (25)	RCT	Migraine	ICHD-2	59 (30/29)	/	/	4 weeks	Shaoyang points	Non-acupuncture points	/	A: Bilateral: SJ20, SJ5, GB34, GB40	Deqi, lifting and inserting twisting 1 time/15 min, 10s/acupoint, 5 times a week, rest 2d between courses, 30 min	TCD	The times of attack, lasting time, accompanying symptoms, and the intensity of attack
											B: Non-acupuncture points next to the meridian points taken by group A			
Zhang et al. (2020), China (50)	Controlled trial	Menstrual migraine (MM)	ICHD-3	55 (31/24)	/	All female	3 months	MM: A1: Real Acupuncture A2: SA	HC	/	A1: Bilateral: GB20, GB8, SP6, PC6, LR3 A2: Non-acupuncture points	27 or (27 ± 6) sessions	fMRI (FC)	Headache attacks, headache intensity (VAS)
Xu et al. (2023), China (80)	Controlled trial	MWoA	ICHD-2	39 (19/20)	A:29.05 ± 7.10	A:2/17	4 weeks	Acupuncture	HC	/	Affected side: SJ23, GB8, EX-HN5, GB20, bilateral: LI4, LR3, GB41, GB34, SJ5	Deqi, twist <90°, 60 times/min, 3 times a week, 30 min	fMRI (DC/GT-LS-BFNA)	VAS
					B:28.35 ± 6.96	B:2/18								
Yu et al. (2023), China (41)	Controlled trial	MWoA	ICHD-2	166 (116/50)	A:40 ± 4	A:47/69	12 weeks	Acupuncture	HC	/	DU20, DU24	1–1.2 inch, 10–20°angle, twist 2–3 min, 180–300 r/min, 2 times, Deqi, once a day, 5 days a week, 45 min	DT-MRI	/
					B:41 ± 5	B:23/27								
Zhang et al. (2022), China (81)	Controlled trial	MWoA	ICHD-3 beta	98 (54/44)	A:37.44 ± 10.64	A:11/43	4 weeks	Acupuncture	HC	/	DU20, DU24, bilateral: GB13, GB8, GB20	Deqi, three times a week, 30 min	rs-fMRI (VMHC)	Number of days of migraine attack, VAS, HIT-6, MIDAS, BAI, BDI
					B:37.57 ± 11.44	B:9/35								
Xu et al. (2023), China (40)	Controlled trial	MWoA	IHS (version not mentioned)	30 (20/10)	A:28.9 ± 6.94	A:2/18	4 weeks	Acupuncture	HC	/	Affected side: SJ23, GB8, EX-HN5, GB20, bilateral: LI4, LR3, GB41, GB34, SJ5	Deqi, three times a week, 30 min	DTI (AFQ)	Headache time, VAS
					B:31.8 ± 8.80	B:2/8								

RCT, randomized controlled trial; MWoA, migraine without aura; IHS, International Headache Society; EA, electroacupuncture; FDG-PET/CT, fluorodeoxyglucose positron emission tomography/computed tomography; VAS, Visual Analogue Scale; SA, sham acupuncture; ICHD, International Classification of Headache Disorders; HC, healthy controls; fMRI, functional magnetic resonance imaging; FC, functional connectivity; ALFF, amplitude of low-frequency fluctuations; ReHo, regional homogeneity; HIT-6, Headache Impact Test-6; HRSD-17, Hamilton Rating Scale for Depression-17; HRSA-14, Hamilton Rating Scale for Anxiety-14; fTCD, functional transcranial Doppler sonography; DTI, diffusion tensor imaging; PSQI, Pittsburgh Sleep Quality Index; ICA, independent component analysis; VA, verum acupuncture; WT, waiting-list; SAS, Self-Rating Anxiety Scale; SDS, Self-Rating Depression Scale; SBA, seed-based analysis; NAP, non-acupoints; MRS, magnetic resonance spectroscopy; CM, chronic migraine; SCA, seed-based correlation analysis; RA, real acupuncture; MMDs, monthly migraine days; MSQ, Migraine-Specific Quality of Life Questionnaire; TA, true acupuncture; BDI-II, Beck Depression Inventory-II; BAI, Beck Anxiety Inventory; MoCA, Montreal Cognitive Assessment; DEC, dynamic effective connectivity; GAD-7, Generalized Anxiety Disorder 7-item scale; dFNC, dynamic functional network connectivity; NRS, Numerical Rating Scale; DC, degree centrality; GT-LS-BFNA, graph theory-based large-scale brain functional network analysis; DT-MRI, diffusion tensor-magnetic resonance imaging; VMHC, voxel-mirrored homotopic connectivity; MIDAS, Migraine Disability Assessment; AFQ, automated fiber quantification.

#### Participants

A total of 1,347 patients and 1,063 HCs were included in this review. All included studies utilized the diagnostic criteria for migraine established by the IHS for participant enrollment. Specifically, 17 studies adopted the International Classification of Headache Disorders (ICHD)-2 version developed by the IHS, 8 studies employed unspecified versions of the ICHD, and 15 studies applied the ICHD-3 version, among which 6 studies utilized the beta version of ICHD-3. 30 studies recruited patients with migraine without aura (MwoA) according to the IHS criteria. 5 studies were unspecified for migraine subtypes, 2 recruited chronic migraine (CM) patients, 2 enrolled menstrual migraine (MM) patients, and 1 focused on non-menstrual migraine patients (Figure 2a). All the studies specify the gender ratio of the included subjects except two studies (25, 26).

In addition, 29 out of 40 studies (72.5%) had baseline HCs and described the age and gender demographic characteristics of the HCs. Among these, 26 studies (65.0%) presented the complete age distribution and mean values of the HCs. 2 studies (5.0%) mentioned that the age of the HCs was matched to that of the observation group, while 1 study (2.5%) did not provide information regarding the age of the HCs. Additionally, 27 studies (67.5%) quantitatively described the gender composition of men and women in the HCs. 1 study (2.5%) stated that the gender composition of the HCs was consistent with that of the observed group, and 1 study (2.5%) did not mention the gender information of the HCs.

#### Acupuncture intervention

32 studies focused on the preventive efficacy of acupuncture for the treatment of migraines; 8 studies primarily explored the immediate efficacy of acupuncture for migraines. The duration of acupuncture treatment courses varied significantly across studies, ranging from 60 s (1 min) to 12 weeks. The needle retention time in the included studies varied from 60 s (1 min) to 45 min, with 30 min being the most frequently reported duration. By summarizing the selection of acupoints for each study, the top five used acupoints were summarized as GB20 (Fengchi, *n* = 21), SJ5 (Waiguan, *n* = 21), GB34 (Yanglingquan, *n* = 18), GB8 (Shuaigu, *n* = 16), and LR3 (Taichong, *n* = 14). 27 studies documented the occurrence of Deqi. 29 studies employed MA, 11 studies used EA. The included studies involved acupuncturists with diverse levels of experience and qualifications. Most practitioners were licensed acupuncturists with substantial clinical experience, ranging from 3 to over 20 years. Several studies specifically mentioned that acupuncturists had specialized training or were registered with relevant health authorities, such as the Ministry of Health of the People’s Republic of China or the National Healthcare Council. Acupuncture treatment sessions could be found in Figure 2b.

#### The controlled intervention

The included literature was categorized into two types of controls, a baseline healthy control group and an intervention control. The interventions included sham acupuncture, and non-specific acupoint controls. Among these, sham acupuncture involved needling at non-acupuncture points (e.g., non-meridian and non-acupoints sites). The non-specific acupoint control group received needling at acupoints unrelated to migraine pathophysiology (e.g., SP3 [Taibai] or GB37 [Guangming]), which were selected based on standardized protocols to exclude therapeutic specificity for migraine.

#### Design and analysis techniques in neuroimaging research

The sample sizes of the included studies ranged from 10 to 143, with 18 studies involving sample sizes greater than or equal to 30. Notably, only one study explicitly referred to sample size calculations. In terms of methodological rigor, the majority of studies (82.5%) reported approval by an ethics committee, demonstrating adherence to ethical standards in human research. However, only a small proportion of studies (17.5%) explicitly mentioned the use of blind testing, while the remaining studies did not report on blinding procedures. Furthermore, less than half of the studies (37.5%) completed enrollment in a clinical trial network, indicating potential gaps in transparency and reproducibility. Thirty-two studies reported clinical outcome measures, the details of which can be found in Figure 2c. The primary clinical outcome indicator was Visual Analog Scale (VAS) (27 studies) and the secondary indicator was headache intensity (15 studies). A substantial number of articles have established correlations between clinical outcome measures and imaging presentations (e.g., functional connectivity [FC], the Amplitude of Low-Frequency Fluctuations [ALFF]) predominantly with respect to VAS, headache intensity, and the number of headache days. Among the 40 studies, the majority employed controlled trial designs (22 studies, 55%), followed by randomized controlled trials (RCTs) (16 studies, 40%). A small proportion of studies (2 studies, 5%) utilized pilot study designs to explore the feasibility and preliminary efficacy of interventions (Figure 2d).

In terms of imaging conditions, 31 articles imposed certain restrictions. Among them, 19 articles stipulated that the imaging procedures should be performed either between migraine attacks or after pain relief, and 7 articles were related to the menstrual cycle. The remaining 9 articles did not mention any limitations regarding scan time. 33 articles systematically documented methodological specifications pertaining to scanning procedures. Postural requirements were specified in 25 studies, with head immobilization being the most frequently mandated condition (*n* = 18), followed by supine positioning (*n* = 10). 26 studies required that the eyes be closed during the scanning process, and 17 required the ears to be plugged, among which 14 required audio—visual closure. 20 studies required the subjects to remain awake and conscious during the scanning process. Additionally, in 9 studies, the subjects were instructed to be as relaxed and quiet as possible, and in 8 studies, the subjects were instructed to minimize thinking activities. Moreover, in 9 studies, the subjects were instructed to rest before the scan. Environmental controls were implemented in 4 studies, primarily involving light attenuation and acoustic isolation of the scanning environment. A singular study required the subjects to be scanned with their eyes open.

A total of 28 studies utilized fMRI, including 1 task-state fMRI and 1 study combining fMRI with DTI. Additionally, 5 studies assessed cerebral blood flow using TCD, 2 studies applied DTI (including 1 using Automated Fiber Quantification, AFQ), 3 studies employed MRS (1 focused on hydrogen-MRS), and 2 studies conducted metabolic imaging with FDG-PET/CT. Among the fMRI studies, 8 applied the ALFF and its variants (e.g., zALFF, dALFF). Of these, 2 studies integrated ALFF with Regional Homogeneity (ReHo), 1 combined ALFF with Dynamic Effective Connectivity (DEC), 1 linked ALFF to Degree Centrality (DC), and 1 utilized ALFF within machine learning frameworks. ReHo was employed in 4 studies, while machine learning integrated with fMRI features (e.g., FC) was implemented in 1 study. Independent Component Analysis (ICA) was used in 3 studies, including 1 combined with Seed-Based Analysis (SBA) and another with Spectral Clustering Analysis (SCA). Other methods included Functional Connectivity (FC; 5 studies), DC with Graph Theory-Based Large-Scale Brain Functional Network Analysis (1 study), SBA (1 study), Seed-to-voxel resting-state functional connectivity (rsFC; 1 study), Voxel-Mirrored Homotopic Connectivity (VMHC; 1 study), and Dynamic Functional Network Connectivity (dFNC; 1 study). Please see Figures 2e,f for details.

### Neuroimaging studies on the immediate efficacy of acupuncture for migraine

**Figure 3:**
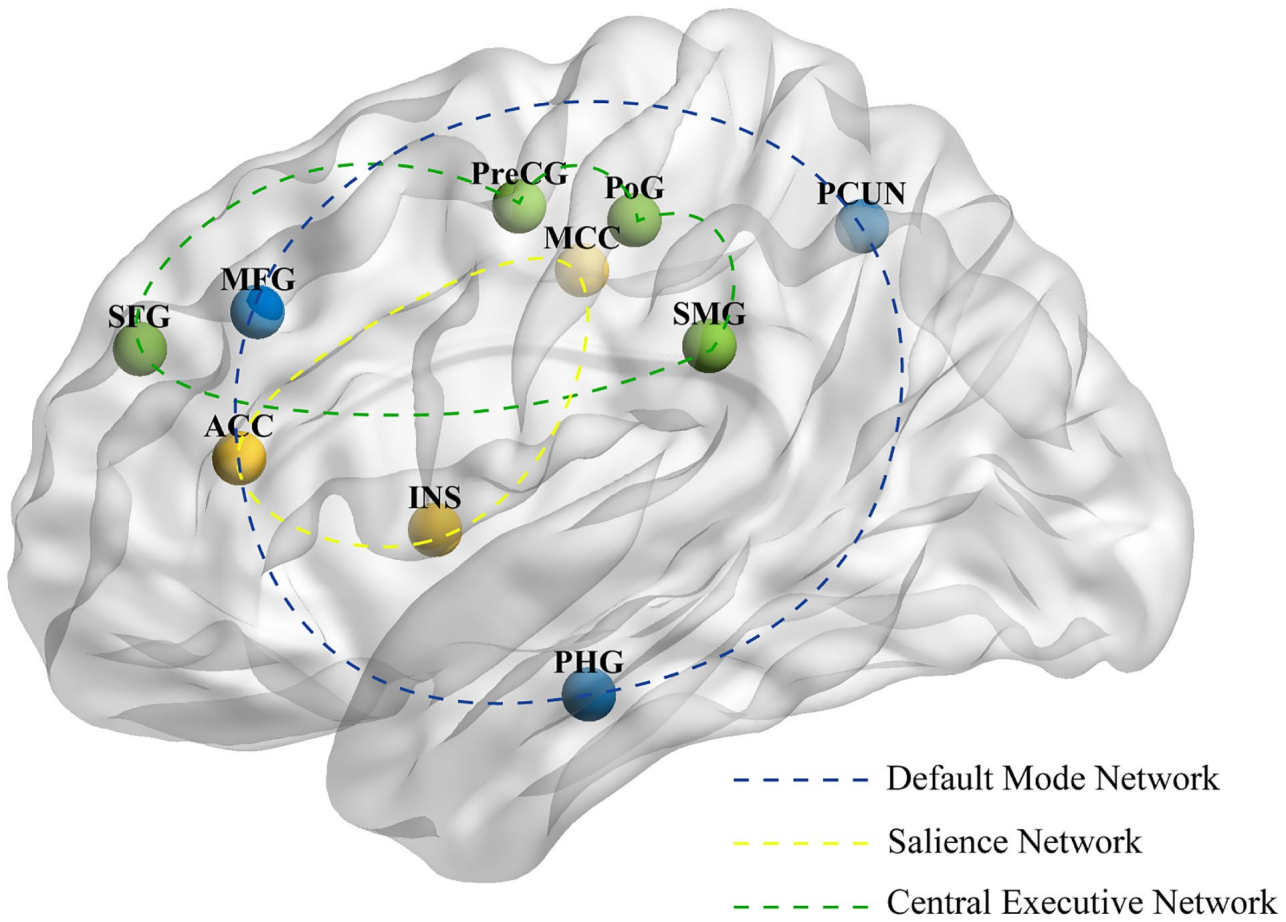
The primary alterations in brain regions and networks induced by immediate acupuncture. PCUN, precuneus; MFG, middle frontal gyrus; PoG, postcentral gyrus; PHG, parahippocampal gyrus; MCC, middle cingulate cortex; INS, insula; SFG, superior frontal gyrus; PreCG, precentral gyrus; ACC, anterior cingulate cortex; SMG; supramarginal gyrus.

Eight studies investigated the immediate neuroimaging effects of acupuncture on migraine. The most frequently reported brain region changes associated with immediate acupuncture in migraine patients are located at the middle frontal gyrus (MFG) (6 studies), precuneus (5 studies), postcentral gyrus (5 studies), parahippocampal gyrus (3 studies), and insula (2 studies). Detailed imaging results for each study are shown in Supplementary material, and the main brain regions and networks showing changes are illustrated in Figure 3.

#### Potential neural mechanisms underlying immediate acupuncture relief for migraine headache

Two FDG-PET/CT studies investigated acute attack of MwoA. The first compared specific Shaoyang and Yangming meridian points, revealing that Shaoyang meridian acupuncture induced greater metabolic changes in pain-related brain regions, including the orbitofrontal cortex, insula, parahippocampus, and hippocampus (22). The second study compared non-specific Shaoyang meridian acupoints with non-meridian points, revealing distinct cerebral metabolic patterns between groups, though analgesic effects showed no significant difference (27). Four studies (28–31) explored neuroimaging changes induced by acupuncture at GB41 (Zulinqi). One study (30) demonstrated enhanced FC in pain-related regions, including the central gyrus, parahippocampal gyrus, cingulate gyrus, and supramarginal gyrus following GB41 stimulation. Additionally, two EA (32, 33) studies stimulate GB8, with one revealing immediate reversal of FC between right hippocampal subregions and the parietal lobe, suggesting modulation of higher cognitive functions and pain perception.

#### Potential neural mechanisms underlying immediate acupuncture relief for migraine non-headache symptoms

Two brain metabolism studies (22, 27) reported changes in the middle temporal cortex (MTC) following acupuncture treatment for migraine. Notably, one study observed increased MTC metabolism after stimulating specific Shaoyang meridian acupoints, while the other found decreased MTC metabolism following non-specific Shaoyang meridian acupoint stimulation. In a controlled trial comparing acupuncture at GB41 between migraine patients and HCs (31), interaction effect analysis revealed that the immediate effect had a differential impact on ReHo in the right lingual gyrus.

### Imaging studies on the preventive efficacy of acupuncture treatment for migraine

**Figure 4:**
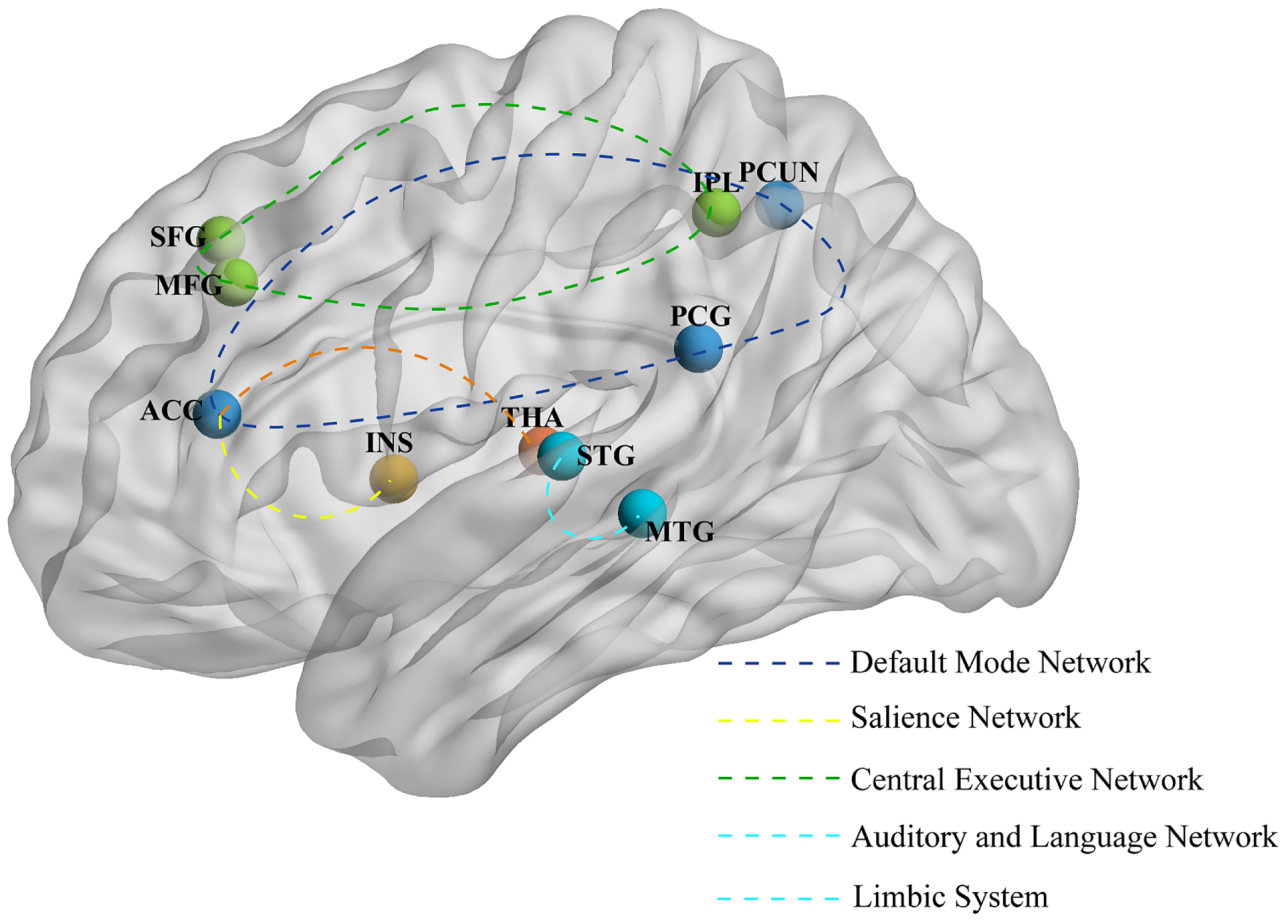
The primary alterations in brain regions and networks induced by preventive acupuncture. ACC, anterior cingulate cortex; PCUN, precuneus; MFG, middle frontal gyrus; IPL, inferior parietal lobule; STG, superior temporal gyrus; THA, thalamus; INS, insula; SFG, superior frontal gyrus; MTG, middle temporal gyrus; PCG, posterior cingulate gyrus.

Thirty-two studies investigated the preventive neuroimaging effects of acupuncture treatment for migraines. The most frequently reported brain region changes associated with preventive acupuncture in migraine patients are located at the precuneus (8 studies), anterior cingulate cortex (ACC) (6 studies), MFG (6 studies), thalamus (6 studies) and inferior parietal lobule (5 studies). Detailed imaging results for each study are shown in Supplementary material, and the main brain regions and networks showing changes are illustrated in Figure 4.

#### Potential neural mechanisms underlying acupuncture treatment for migraine headache prevention

Five TCD studies (25, 26, 34–36) have demonstrated the modulatory effects of acupuncture on cerebrovascular function in migraine patients. Specifically, Three studies (25, 26, 36) targeting Shaoyang meridian points showed improved cerebral blood flow velocity post-treatment, characterized by reduced peak systolic velocity in the middle cerebral artery (MCA), posterior cerebral artery (PCA), vertebral artery (VA), and basilar artery (BA), with significant differences in PCA, right vertebral artery (RVA), and BA compared to the non-acupuncture group. Three MRS studies (37–39) revealed increased NAA/Cr (N-acetylaspartate/creatine) ratios in the bilateral thalamus and periaqueductal gray matter (PAG) following acupuncture treatment. Furthermore, Two DTI studies (40, 41) demonstrated that acupuncture improved white matter integrity in migraine patients, particularly in the right corticospinal tract, right superior longitudinal fasciculus, left central gyrus, and the genu, body, and splenium of the corpus callosum.

Based on fMRI studies, multiple neuroimaging studies have shown significant modulation of brain activity following acupuncture treatment. Specifically, increases in ALFF and ReHo values were observed in key brain regions associated with pain processing (such as the thalamus, anterior cingulate gyrus, and insula) (42–44), emotional regulation of pain (such as the precuneus and cerebellum) (43–45), cognitive evaluation of pain (such as the MFG and angular gyrus) (42, 45, 46) and descending pain modulatory system [DPMS] (such as the brainstem, including the rostral ventromedial medulla/trigeminocervical complex) (43, 47). Conversely, decreased ALFF and ReHo values were found in regions such as the precuneus, posterior cingulate gyrus, hippocampus, and inferior frontal gyrus (42–44). Our study found that FC between the PAG and the rostral anterior cingulate cortex (rACC) was significantly increased in migraine patients after acupuncture treatment, suggesting that damage to the downstream pain pathway is associated with the neuropathophysiology of migraine (48). Additionally, in a FC analysis using the precuneus as a seed region, it was found that the rs-fc between the precuneus and key regions of the reward system, cognitive control network, and DPMS was significantly enhanced after acupuncture treatment (49). Notably, three studies (46, 50, 51) focused on female migraine patients. Among them, a recent study (51), using resting-state blood oxygen level-dependent fMRI, found that the ALFF and DC values decreased in young female patients with non-menstrual MWoA after acupuncture treatment. Another study on menstrual migraine patients revealed that the FC between the precuneus and the middle frontal gyrus, as well as between the precuneus and the caudate nucleus, significantly increased after acupuncture at specific acupoints compared to before treatment (50).

#### Potential neural mechanisms underlying acupuncture treatment for migraine non-headache symptoms prevention

Acupuncture treatment for migraine can induce changes in brain activity, including decreased ReHo or ALFF values in the superior temporal gyrus (46), fusiform gyrus (42, 51), inferior temporal gyrus (43, 51), and middle occipital gyrus (47). An FC study revealed significant enhancement of connectivity between the right amygdala or middle cingulate cortex (MCC) and the left temporal lobe, as well as between the right MCC and the right superior temporal gyrus (52). Furthermore, another study demonstrated increased FC between the temporal lobe and the ACC, bilateral superior medial gyrus, and bilateral precuneus following acupuncture treatment (53).

## Discussion

This review included 40 articles exploring the central mechanisms of acupuncture using neuroimaging techniques from both Chinese and English databases. Among these, 32 articles primarily investigated the preventive effects of acupuncture on neuroimaging in migraine, while 8 articles focused on the immediate effects. By systematically reviewing the 40 neuroimaging studies on acupuncture treatment for migraine, this paper analyzes the methodological issues and research findings, providing an in-depth understanding of the potential neuroimaging mechanisms underlying acupuncture treatment for migraine. In the following sections, we will discuss three aspects: the design status of existing studies, the analysis of current neuroimaging results, and prospects for future research.

### The design status of existing studies

Among the included literatures, only 16 studies were RCT designed neuroimaging studies, while the rest were non-RCT designed. To ensure the effectiveness of the acupuncture treatment protocols applied in neuroimaging trials for migraine treatment, it is recommended to conduct more neuroimaging studies with randomized designs to improve the level of evidence. Notably, some researchers have suggested that statistical efficacy can be achieved with 12 to 15 subjects per group in fMRI studies (54, 55). A total of five in the included literature were below 12 subjects per group. These small sample sizes may affect the reliability and reproducibility of expected effect sizes in neuroscience (56). In addition, existing studies have shown that more women than men suffer from migraine, but only three studies have specifically targeted female patients. Therefore, future studies should prioritize identifying and exploring disease-specific targets that may be unique to female migraineurs.

Focusing on acupuncture techniques, a total of 8 studies did not provide detailed information on the qualifications or operational experience of the acupuncturists. Variability in techniques among different acupuncturists makes it difficult to precisely quantify the intensity of stimulation. Therefore, to ensure the reproducibility and consistency of research findings, researchers should strictly adhere to the STRICTA guidelines (57), establish detailed acupuncture protocols, and provide standardized training for acupuncturists before each trial. This approach will effectively ensure that all participants receive consistent stimulation, thereby enhancing the scientific rigor and reliability of the studies. According to traditional Chinese medicine theory, the sensation of Deqi plays a central role in the effectiveness of acupuncture. It is encouraging to note that at least half of the studies have reported this phenomenon. Previous neuroimaging studies (58) have elucidated the brain’s response to the Deqi sensation, highlighting its significant importance in the therapeutic effects of acupuncture.

Regarding the timing of imaging scans, 19 articles specified that imaging procedures should be conducted during the interictal phase or after pain relief, while only 2 studies focused on acute migraine attacks. The unpredictability of migraine attacks, along with the pain and neurological symptoms, as well as the time-consuming and noisy nature of fMRI scans, have affected patients’ coordination and compliance with clinical research. These limitations make it challenging to conduct neuroimaging studies during migraine attacks, resulting in a relatively limited number of related studies. Future studies should strive to overcome these difficulties and explore novel imaging technologies or more flexible research designs that are better suited for the acute attack phase.

### The analysis of current neuroimaging results

The brain region changes caused by immediate acupuncture treatment mainly include the middle frontal gyrus, precuneus, and postcentral gyrus, and the major brain networks involved are the DMN, SN, and CEN. Chronic pain, as a complex sensory experience, is closely related to the modulation of these networks (59). DMN is involved in self-referential processing, consciousness, awareness, mind-wandering, episodic memory, and the manipulation of semantic knowledge (60, 61). CEN supports higher-level cognitive processes, including the regulation of emotions, behaviors, and attentional control (62, 63). SN is crucial for stimulus detection and the allocation of attentional resources (64). These networks play a crucial role in the regulation of cognition, emotions, and behaviors.

The brain region changes caused by preventive acupuncture treatment mainly include the precuneus, ACC, and middle frontal gyrus. The major brain networks involved are the DMN, SN, CEN, and DPMS, among others. The imbalance between the trigeminal pain ascending pathway and the DPMS is a key anatomical pathway for understanding migraine. Effective acupuncture treatment can help restore this balance (48). In addition, acupuncture can also improve the rs-FC of the frontoparietal network in migraine patients, thereby alleviating headache symptoms by enhancing cognitive adaptation and coping processes (49). In summary, acupuncture treatment effectively alleviates pain symptoms by actively modulating the functions of brain regions and networks.

Acupuncture not only alleviates the headache symptoms of migraine but also mitigates non-headache symptoms such as photophobia and phonophobia. The literature we reviewed indicates that acupuncture can modulate brain regions including the superior temporal gyrus (STG), occipital gyrus, and fusiform gyrus. Notably, multiple studies have demonstrated that the STG is involved in enhanced multisensory processing in migraine patients, particularly in audition, vision, and olfaction (65–67). The occipital cortex, as the core area for visual perception, and the temporo-occipital region, with its complex neural connections involved in integrating visual, auditory, and tactile information, jointly support multisensory processing (68, 69). Additionally, previous studies have shown that migraine patients exhibit reduced activity and neural synchrony in the occipital lobe, which is generally associated with visual aura (70, 71).

### The prospects for future research

Currently, neuroimaging studies on acupuncture are confronted with several methodological limitations that urgently need to be addressed. To enhance the scientific rigor of these studies, we emphasize the necessity for future research to: (1) strictly design and report sham acupuncture control groups; (2) provide detailed documentation of the implementation of blinding procedures; and (3) list clinical trial registration information. Despite these improvements, existing neuroimaging research on acupuncture continues to face multifaceted challenges. First, there is a paucity of research on different migraine subtypes (e.g., migraine with aura), particularly from a neuroimaging perspective, and even fewer studies exploring the mechanisms of acupuncture in relation to these subtypes. Second, existing research has predominantly focused on the interictal phase of migraine, while studies on other critical phases (e.g., pre-ictal, ictal, and post-ictal) remain relatively scarce. These phases may better reflect the dynamic regulatory effects of acupuncture on migraine. Additionally, migraine is not only characterized by headache but is often accompanied by non-headache symptoms, such as autonomic symptoms (e.g., nausea, vomiting) and cortical spreading depression (CSD). However, research investigating the mechanisms of acupuncture from these perspectives is still insufficient. In the future, more comprehensive studies should be conducted across multiple dimensions, encompassing different migraine subtypes, phases, and non-headache symptoms. Crucially, future research should track neuroimaging changes and clinical outcomes following acupuncture in patients to identify objective neural correlates of sustained therapeutic effects and individual differences in response. To further elucidate acupuncture’s mechanisms and provide a more robust scientific basis for migraine treatment, integrating advanced neuroimaging techniques, such as transcriptomic and molecular imaging, alongside animal studies will be essential.

## Limitations

This study has several limitations. First, the inclusion of various imaging modalities and analytical approaches made it difficult to conduct a comprehensive quantitative meta-analysis. Second, the limited number of high-quality studies published may have constrained our findings, and considering the instability arising from small sample sizes (as discussed earlier), the results should be interpreted with caution.

## Conclusion

This systematic review reveals that the brain regions involved in acupuncture treatment for migraine headache include the DMN, CEN, SN and DPMS, which are related to pain sensation, emotion and modulation. Future research could prioritize meticulously designed, high-quality, multimodal neuroimaging studies to further elucidate the neuroimaging evidence for acupuncture in migraine treatments from different aspects.

## Data Availability

The original contributions presented in the study are included in the article/Supplementary material, further inquiries can be directed to the corresponding authors.
