# Supplementary material for: Acupuncture for migraine: a literature review of neuroimaging studies
Source: Front Neurol. 2025 Jun 25;16:1601554. doi: 10.3389/fneur.2025.1601554 (PMC12237655; doi:10.3389/fneur.2025.1601554)
Supplement: Supplementary file 1 [file Data_Sheet_1.DOCX]

Supplementary Material

# Search strategies

| **Search strategies** | | | |
| --- | --- | --- | --- |
| **Databases** | **Coverage** | **Searches** | **Hits** |
| PubMed Database | Date of inception -November 19, 2024 | #1 "Migraine Disorders"[Mesh]  #2 (Migraine Disorders[Title/Abstract]) OR (migraine[Title/Abstract])  #3 #1 OR #2  #4 (("Acupuncture"[Mesh]) OR "Acupuncture Therapy"[Mesh]) OR "Acupuncture Analgesia"[Mesh]  #5 ((((((Acupuncture Therapy[Title/Abstract]) OR (Acupuncture[Title/Abstract])) OR (Acupuncture Points[Title/Abstract])) OR (acupressure[Title/Abstract])) OR (electroacupuncture[Title/Abstract])) OR (meridians[Title/Abstract])) OR (moxibustion[Title/Abstract])  #6 #4 OR #5  #7 (("Neuroimaging"[Mesh]) OR "Magnetic Resonance Imaging"[Mesh]) OR "Positron-Emission Tomography"[Mesh]  #8 (((((((((((((((neuroimaging[Title/Abstract]) OR (magnetic resonance imaging[Title/Abstract])) OR (Positron-Emission Tomography[Title/Abstract])) OR (functional magnetic resonance imaging[Title/Abstract])) OR (fMRI[Title/Abstract])) OR (Diffusion Tensor Imaging[Title/Abstract])) OR (functional image[Title/Abstract])) OR (DTI[Title/Abstract])) OR (structural magnetic resonance imaging[Title/Abstract])) OR (sMRI[Title/Abstract])) OR (functional near-infrared spectroscopy[Title/Abstract])) OR (fnirs[Title/Abstract])) OR (electroencephalography[Title/Abstract])) OR (magnetic resonance spectroscopy[Title/Abstract])) OR (magnetoencephalography[Title/Abstract])) OR (transcranial Doppler[Title/Abstract])  #9 #7 OR #8  #10 #3 AND #6 AND #9 | **60** |
| EMBASE Database | Date of inception -November 19, 2024 | #1 'migraine'/exp/mj  #2 'migraine disorders':ti,ab,kw OR 'migraine':ti,ab,kw  #3 #1 OR #2  #4 'acupuncture'/exp OR 'acupuncture therapy'/exp OR 'acupuncture analgesia'/exp  #5 'acupuncture therapy':ti,ab,kw OR 'acupuncture':ti,ab,kw OR 'acupuncture analgesia':ti,ab,kw OR 'acupuncture points':ti,ab,kw OR 'acupressure':ti,ab,kw OR 'electroacupuncture':ti,ab,kw OR 'meridians':ti,ab,kw OR 'moxibustion':ti,ab,kw  #6 #4 OR #5  #7 'neuroimaging'/exp OR 'magnetic resonance imaging'/exp OR 'positron-emission tomography'/exp  #8 'neuroimaging':ti,ab,kw OR 'magnetic resonance imaging':ti,ab,kw OR 'positron-emission tomography':ti,ab,kw OR 'functional magnetic resonance imaging':ti,ab,kw OR 'fmri':ti,ab,kw OR 'diffusion tensor imaging':ti,ab,kw OR 'functional image':ti,ab,kw OR 'dti':ti,ab,kw OR 'structural magnetic resonance imaging':ti,ab,kw OR 'smri':ti,ab,kw OR 'functional near-infrared spectroscopy':ti,ab,kw OR 'electroencephalography':ti,ab,kw OR 'magnetic resonance spectroscopy':ti,ab,kw OR 'magnetoencephalography':ti,ab,kw OR 'transcranial doppler':ti,ab,kw OR 'fnirs':ti,ab,kw  #9 #7 OR #8  #10 #3 AND #6 AND #9 | **139** |
| Cochrane Library | Date of inception -November 19, 2024 | #1 MeSH descriptor: [Migraine Disorders] explode all trees  #2 (migraine disorders):ti,ab,kw OR (migraine):ti,ab,kw #3 #1 OR #2 #4 MeSH descriptor: [Acupuncture] explode all trees #5 MeSH descriptor: [Acupuncture Therapy] explode all trees #6 MeSH descriptor: [Acupuncture Analgesia] explode all trees #7 (acupuncture therapy):ti,ab,kw OR (acupuncture):ti,ab,kw OR (acupuncture analgesia):ti,ab,kw OR (acupuncture points):ti,ab,kw OR (acupressure):ti,ab,kw OR (electroacupuncture):ti,ab,kw OR (meridians):ti,ab,kw OR (moxibustion):ti,ab,kw #8 #4 OR #5 OR #6 OR #7 #9 MeSH descriptor: [Neuroimaging] explode all trees #10 MeSH descriptor: [Magnetic Resonance Imaging] explode all trees #11 MeSH descriptor: [Positron-Emission Tomography] explode all trees  #12 (neuroimaging):ti,ab,kw OR (magnetic resonance imaging):ti,ab,kw OR (positron-emission tomography):ti,ab,kw OR (functional magnetic resonance imaging):ti,ab,kw OR (fmri):ti,ab,kw OR (diffusion tensor imaging):ti,ab,kw OR (functional image):ti,ab,kw OR (dti):ti,ab,kw OR (structural magnetic resonance imaging):ti,ab,kw OR (smri):ti,ab,kw OR (functional near-infrared spectroscopy):ti,ab,kw OR (electroencephalography):ti,ab,kw OR (magnetic resonance spectroscopy):ti,ab,kw OR (magnetoencephalography):ti,ab,kw OR (transcranial doppler):ti,ab,kw OR (fnirs):ti,ab,kw  #13 #9 OR #10 OR #11 OR #12  #14 #3 AND #8 AND #13 | **49** |
| Web of science | Date of inception -November 19, 2024 | TS=((‘migraine disorders’ OR ‘migraine’) AND (‘acupuncture*’ OR ‘acupuncture-moxibustion’ OR ‘meridian*’ OR ‘acupoint*’ OR ‘acupuncture plus moxibustion’ OR ‘electroacupuncture’ OR ‘acupressure’) AND (‘neuroimaging’ OR ‘magnetic resonance imaging’ OR ‘Positron-Emission Tomography’ OR ‘functional magnetic resonance imaging’ OR ‘fMRI’ OR ‘functional image’ OR ‘Diffusion Tensor Imaging’ OR ‘DTI’ OR ‘structural magnetic resonance imaging’ OR ‘sMRI’ OR ‘functional near-infrared spectroscopy’ OR ‘electroencephalography’ OR ‘magnetic resonance spectroscopy’ OR ‘magnetoencephalography’ OR ‘fnirs’OR ‘transcranial doppler’)) | **87** |
| China National Knowledge Infrastructure (CNKI, Chinese Database) | Date of inception -November 19, 2024 | SU=(针+针灸+针刺+电针+针刺疗法+针刺治疗)*(偏头痛)*(神经成像+磁共振成像+正电子发射断层扫描+功能性磁共振成像+fMRI+功能性图像+弥散张量成像+DTI+结构性磁共振成像+sMRI+功能性近红外光谱成像+脑电图+磁共振波谱+磁脑图+fMRI+经颅多普勒) | **109** |
| Chinese Biomedical Literature Database  (CBM, Chinese Database) | Date of inception -November 19, 2024 | 1) "偏头痛"[不加权:扩展]  2) "针灸疗法"[不加权:扩展]  3) "针"[常用字段:智能] OR "针灸"[常用字段:智能] OR "针刺"[常用字段:智能] OR "电针"[常用字段:智能] OR "针刺治疗"[常用字段:智能]  4) (#2) OR (#3)  5) (#1) AND (#4)  6) "神经成像"[不加权:扩展]  7) "磁共振成像"[常用字段:智能] OR "正电子发射断层扫描"[常用字段:智能] OR "功能性磁共振成像"[常用字段:智能] OR "fMRI"[常用字段:智能] OR "功能性图像"[常用字段:智能] OR "弥散张量成像"[常用字段:智能] OR "DTI"[常用字段:智能] OR "结构性磁共振成像"[常用字段:智能] OR "sMRI"[常用字段:智能]  8) "功能性近红外光谱成像"[常用字段:智能] OR "脑电图"[常用字段:智能] OR "磁共振波谱"[常用字段:智能] OR "磁脑电"[常用字段:智能] OR "fMRI"[常用字段:智能] OR "经颅多普勒"[常用字段:智能]  9) (#6) OR (#7) OR (#8)  10) (#9) AND (#5) | **100** |
| Chongqing VIP Database  (VIP, Chinese Database) | Date of inception -November 19, 2024 | 主题:(偏头痛) and 主题:(针 or 针灸 or 针刺 or 电针 or 针刺疗法 or 针刺治疗) and 主题:(神经成像 or 磁共振成像 or 正电子发射断层扫描 or 功能性磁共振成像 or fMRI or 功能性图像 or 弥散张量成像 or DTI or 结构性磁共振成像 or sMRI or 功能性近红外光谱成像 or 脑电图 or 磁共振波谱 or 磁脑图 or fMRI or 经颅多普勒) | **241** |
| Wanfang Database  (WF, Chinese Database) | Date of inception -November 19, 2024 | M=(针+针灸+针刺+电针+针刺疗法+针刺治疗)*(偏头痛)*(神经成像+磁共振成像+正电子发射断层扫描+功能性磁共振成像+fMRI+功能性图像+弥散张量成像+DTI+结构性磁共振成像+sMRI+功能性近红外光谱成像+脑电图+磁共振波谱+磁脑图+fMRI+经颅多普勒) | **48** |

# Neuroimaging Results of Immediate Treatment Effects

| **Author, Year, Country** | **Results** |
| --- | --- |
| Yang 2012, China | **Clinical outcome:** ↓VAS ( Visual Analogue Scale) **PET outcome: TAG vs. MG:** ↑Metabolism in the middle temporal cortex (MTC), orbital frontal cortex (OFC), insula, middle frontal gyrus, angular gyrus, post-cingulate cortex (PCC), the precuneus, and the middle cingulate cortex (MCC) ↓Metabolism in the parahippocampus, hippocampus, fusiform gyrus, postcentral gyrus, and cerebellum **CAG vs. MG:** ↑Metabolism in the MTC, supratemporal gyrus, supramarginal gyrus , and MCC ↓Metabolism in the cerebellum |
| Yang 2014, China | **Clinical outcome:** ↓VAS **PET outcome: AG vs. MG:** ↑Metabolism in the middle frontal gyrus, postcentral gyrus, the precuneus, parahippocampus, cerebellum and MCC ↓Metabolism in the left hemisphere of MTC **SAG vs. MG:** ↑Metabolism in PCC, insula, inferior temporal gyrus, MTC, superior temporal gyrus, postcentral gyrus, fusiform, inferior parietal lobe, superior parietal lobe, supramarginal gyrus, middle occipital lobe, angular and precuneus ↓Metabolism in cerebellum, parahippocampus |
| Liu 2016, China | **FC outcome: Post- VS. pre-treatment (migraine vs. HC):** ↓The FC values in the parahippocampal gyrus, posterior cingulate gyrus and angular gyrus **Post- VS. pre-treatment (migraine):**  ↑The FC values in areas distributed posterior to the central gyrus, parahippocampal gyrus, cingulate gyrus and supramarginal gyrus with varying degrees |
| Ning 2017, China | **Pre- vs. HC:** ↓ALFF (Amplitude of low frequency fluctuations) values in the left calcarine, cuneus, parietal gyrus ↑ALFF values in the right hippocampus, parahippocampal gyrus, insula, middle temporal gyrus and superior temporal gyrus **Post- vs. pre-treatment:** ↓ALFF values in the bilateral precuneus, right inferior parietal lobule and middle frontal gyrus ↑ALFF values in the right precentral and postcentral gyri **Post- vs. HC:** ↓ALFF values in the left precuneus, calcarine, cuneus, superior parietal gyrus, inferior parietal but supramarginal and angular gyri ↑ALFF values in the right precentral, postcentral gyri, hippocampus, middle temporal gyrus and superior temporal gyrus |
| Han 2017, China | **ReHo outcome: Pre-acu vs. HC:** ↓ReHo values in the right middle occipital gyrus, supraoccipital gyrus, cuneus, precuneus, and perisylvian fissure cortex ↑ReHo values in the bilateral superior orbitofrontal gyrus, middle orbitofrontal gyrus, medial orbitofrontal gyrus, rectus gyrus, left medial superior frontal gyrus, and right infraorbital frontal gyrus **Post- VS. pre-treatment (migraine vs. HC):** Brain regions with differences were the right lingual gyrus |
| Ning 2020, China | **ALFF outcome: Pre- vs. HC:** ↑The ALFF oscillations of the hippocampus，para-hippocampal gyrus，and right superior temporal gyrus ↓The ALFF oscillations of the left cuneiform lobe and supratopia back to the other brain areas **Post- vs. pre-treatment:**  ↑The ALFF oscillations of the right central anterior and posterior gyrus ↓The ALFF oscillations of the right precuneus, middle frontal gyrus, etc. and other brain area **Post- vs. HC:** ↑The ALFF oscillations of the right central anterior gyrus, central posterior gyrus and other brain areas ↓The ALFF oscillations of the left cuneiform and precuneus |
| Wei 2022, China | **FC outcome:**  **Pre- vs. HC:** ↓FC values between the right ACC （anterior cingulate cortex） and right middle occipital gyrus, left caudate nucleus, left medial superior frontal gyrus, left anterior central gyrus, right middle frontal gyrus and left middle frontal gyrus in the patient group **Post- vs. pre-treatment:** ↑The FC values between left ACC and left pars triangularis, left and right middle frontal gyrus, the right ACC and left pars triangularis, left superior frontal gyrus, left and right middle frontal gyrus **Task-related fMRI-BOLD outcome:** ↑BOLD values in left pars triangularis of frontal inferior gyrus(BA46), left anterior central gyrus, left posterior gyrus, right Rolandic operculum and right supramarginal gyrus **Correlation:** The BOLD values in the dlPFC (dorsolateral prefrontal cortex) were negatively correlated with the duration of disease |
| Luo 2022, China | **FC outcome: PMa vs. HCs and PMb:** ↓Right dorsal anterior insula-right postcentral gyrus ↑Right posterior insula-left precuneus **Correlation:** The FC value of the right postcentral gyrus was negatively correlated with the HRSD (HamiltonRating Scale for Depression) and HRSA (Hamilton Rating Scale for Anxiety) scores The FC value of the left precuneus was positively correlated with VAS score |

**Abbreviations:** PET, Positron Emission Tomography; TAG, traditional acupuncture group; MG, migraine group; CAG, control acupuncture group; AG, acupuncture group; SAG, sham acupuncture group; HC, healthy control; FC, functional connectivity; PM, patients with MWoA; PMa, PM before EA; PMb, PM during EA; fMRI, functional magnetic resonance imaging; BOLD, blood oxygen level-dependent; ReHo, regional homogeneity.

# Neuroimaging Results of **Preventive** Treatment Effects

| **Author, Year, Country** | **Conclude** |
| --- | --- |
| Bäcker 2004, Germany | Overshooting cerebral blood flow velocity and a delayed decline in MCA (middle cerebral artery), PCA (posterior cerebral artery) |
| Zhao 2014, China | **Clinical outcome:** ↓VAS, frequency of migraine attack, number of days with migraine, and HIT-6 (Headache Impact Test-6) score **ReHo outcome: Post- VS. pre-treatment (Active acupuncture):** ↑ReHo in the bilateral ACC, insula, thalamus, supplementary motor area (SMA), superior temporal gyrus (STG), cuneus, lingual gyrus, cerebellum, and brainstem ↓ReHo in the bilateral PCC, middle frontal gyrus (MFG), angular gyrus, precuneus, middle temporal gyrus (MTG), left hippocampus, inferior parietal lobule (IPL), inferior temporal gyrus (ITG), and right postcentral gyrus **Post- VS. pre-treatment (Inactive acupuncture):** ↑ReHo in the left ACC and medial frontal gyrus ↓ReHo in the right MFG **Post- VS. pre-treatment (Active acupuncture vs. Inactive acupuncture):** The active acupoint group showed higher ReHo in the thalamus, ACC, STG, SMA and lower ReHo in the hippocampus, MFG, and MTG than the inactive group **Correlation:** Decrease in the VAS score was significantly related to the increased average ReHo values in the ACC and insula |
| Li 2015, China | **Clinical outcome:** ↓VAS, duration and frequency of migraine attacks **FC outcome: Pre- vs. HC:** ↓FC with the right frontoparietal network (RFPN) in the left precentral gyrus, the left supramarginal gyrus, the left IPL and the left postcentral gyrus **Post- vs. pre-treatment:** ↑FC with the RFPN in the left precentral gyrus, the left IPL, and the left postcentral gyrus **Correlation:** Increased FC of brain regions was negatively correlated with the decrease of VAS scores after treatment |
| Zhang 2016, China | **Clinical outcome:** ↓VAS and the Pittsburgh Sleep Quality Index (PSQI) scores, the duration and frequency **FC outcome: Pre- vs. HC: ↓**FC in the bilateral superior frontal gyrus (SFG), medial frontal gyrus, IPL, ACC, cingulate gyrus (CG), PCC, supramarginal gyrus, precuneus, middle frontal gyrus, inferior frontal gyrus, STG, and MTG **Post- vs. pre-treatment:** ↑FC in the bilateral SFG, medial frontal gyrus, precuneus, IPL, posterior cingulate cortex, CG, STG, MTG, and supramarginal gyrus |
| Li 2016, China | **Clinical outcome: ↓**VAS (VA1, VA2, VA3) **FC outcome: MG vs. HC:** ↑Rs-fc between the ventrolateral periaqueductal gray (PAG) and the bilateral adjacent PAG ↓Rs-fc between the ventrolateral PAG and the bilateral medial prefrontal cortex (mPFC), OFC (orbitofrontal cortex), rostral anterior cingulate cortex (rACC) **Post- VS. pre-treatment:** ↑Rs-fc between ventrolateral periaqueductal gray (vlPAG) and the bilateral MCC and rACC, and left mPFC **VA vs. SA:** ↑ Rs-fc between ventrolateral PAG and the bilateral ventral/anterior mPFC, left middle occipital gyrus/cunues, right middle occipital gyrus/cunues **Correlation:** Negative association between VAS (post-pre) and rs-FC (post-pre) between vlPAG and brain regions including bilateral rACC and MCC, and left SFG, thalamus, putamen, caudate and cerebellum, and right SMA/preSMA and middle frontal gyrus |
| Li 2017, China | **Clinical outcome: ↓**VAS (VA1, VA2, VA3) **ALFF outcome:  MG vs. HC:** ↑ALFF at the left posterior insula and left putamen/caudate ↓ALFF in the bilateral middle occipital cortex/cuneus and bilateral rostral ventromedial medulla (RVM)/trigeminocervical complex (TCC) **Post- vs. pre-treatment:  ↑**Bilateral OFC, bilateral RVM/TCC and bilateral rostral midbrain ↓Left middle occipital cortex/cuneus **VA vs. SA:** ↑Bilateral RVM/TCC |
| Li 2017, China | **Clinical outcome:** ↓VAS and headache frequency (VA1, VA2, VA3) **ICA outcome: MG vs. HC:** ↓Rs-fc between the RFPN with the bilateral precuneus, lingual gyrus, MTG, STG, left fusiform, secondary somatosensory cortex (S2), right cerebellum, inferior occipital gyrus, ITG and cuneus **Post- VS. pre-treatment:** ↓Rr-fc between the RFPN with the right precuneus and left MFG ↑Rs-fc with the bilateral posterior cingulate cortex for RFPN ↑Right precuneus rs-fc with the bilateral rACC/mPFC, ventral striatum, middle/inferior occipital gyrus, cuneus, DLPFC and cerebellum, left VLPFC and right STG **Correlation:** The decrease of rs-fc with the bilateral precuneus, right paracentral gyrus and postcentral gyrus for rFPN was positively associated with a decrease in headache intensity |
| Gu 2018, China | **Clinical outcome:** ↓VAS, duration of headache attacks **MRS outcome: Post- VS. pre-treatment (AG group):** ↑NAA/Cr in the bilateral thalamus **Post- VS. pre-treatment (HC group):** No difference **Correlation:** Correlation between NAA (N-acetylaspartate)/Cr (creatine) and VAS in bilateral thalamus in post-treatment follow-up |
| Zou 2019, China | **Clinical outcome:** ↓Headache attacks, monthly mean/immediate VAS, headache days, acute headache medications **Results: CM vs. HC:** Reductions in the left superior prefrontal gyrus, left precuneus and decreased default mode network (DMN) z-scores within the two regions ↓FC between the right temporal lobe and left ACC, between the right temporal lobe and bilateral precuneus, between the the right temporal lobe and bilateral superior medial gyrus, between the right temporal lobe and bilateral superior prefrontal gyrus, between the right temporal lobe and left temporal lobe **Post- VS. pre-treatment:** ↑FC between the right temporal lobe and left ACC, between the right temporal lobe and bilateral superior medial gyrus, between the right temporal lobe and bilateral precuneus. **Correlation:** Increased z-scores within the L_SPFG and L_PRECUN (left superior prefrontal gyrus and left precuneus) were associated with reduced immediate VAS scores, and increases in z-scores of the L_PRECUN were negatively correlated with reductions in the monthly amount of acute headache medications |
| Tu 2020, China | **MG vs. HC:** The differences were located primarily within the occipital lobe, including both occipital and postoccipital areas (middle occipital gyrus and calcarine); the sensorimotor network, including the parietal and postparietal(IPL) cortices; part of the medial-cerebellum; the cingulo-opercular network, including the anterior-insula, dorsal ACC, medial frontal cortex, and thalamus; DMN, including the angular gyrus, fusiform gyrus, occipital gyrus; the frontal parietal network, including the anterior frontal cortex and ventral lateral PFC **AG vs. SAG vs. MG:** We linked the changes in the connectome-based response to changes in headache frequency and found a significant correlation in AG but not in SAG or MG |
| Yin 2020, China | **Clinical outcome:**  ↓VAS  **zALFF outcome: Post- VS. pre-treatment:** ↓In the right middle occipital gyrus and the left middle occipital gyrus **Correlation:** zALFF value of right middle occipital gyrus positively correlated with the improvement of VAS scores zALFF value of left middle occipital gyrus positively correlated with the relief of mean migraine days zALFF value of right fusiform positively correlated with the duration |
| Tian 2021, China | **Clinical outcome: ↓**SAS (Self-Rating Anxiety Scale), SDS ( Self-Rating Depression Scale), headache intensity **FC outcome: Pre- vs. HCs:** ↓FCs between CG and IPL, CG and insular gyrus (INS) , and MFG and IPL ↑FCs between CG and SFG **Post- VS. pre-treatment: ↓**Amygdala and insula, Amyg and SFG, CG and SFG, Hipp and SFG, and thalamus (Tha) subregions ↑Amygdala and MFG, hippocampus (Hipp) and MFG, Hipp and INS, IPL and INS,IPL and MFG, IPL and SFG, and Tha subregions **Correlation:** FCs between the left Amyg and left MFG, the left Amyg and left SFG correlated with the rate of improvement in headache intensity |
| Liu 2021, China | **Clinical outcome:** ↓Migraine days, VAS, SAS, SDS ↑MSQ ( Migraine-Specific Quality of Life Questionnaire) **ReHo outcome: Pre- vs. HCs:** ↓The ReHo values of the cerebellum **Post- VS. pre-treatment:** ↑The ReHo values of the cerebellum, angular gyrus (after 12 acupuncture sessions) **Correlation:** Positive correlations of the change in ReHo value in the angular gyrus with days of migraine at baseline and with the change in number of days with migraine |
| Zhang 2021, China | **Clinical outcome:** ↓SAS, SDS, VAS, intensity of migraine, frequency of attacks ↑Emotional states **ALFF outcome (After TA treatment):** ↑Right MFG ↓Left ACC, right inferior frontal gyrus **ReHo outcome (After TA treatment):** ↑Right SFG, left cuneus, and right MFG ↓Right STG **ALFF/ReHo outcome (After SA treatment):** ↑ALFF values in the right lingual gyrus and left insula ↑ReHo value in the left insula **Correlation**： Altered ALFF value in the left ACC was positively correlated with the decreases in the SAS, SDS scores in TA |
| Liu 2022, China | **Clinical outcome:** ↓Headache severity (monthly migraine days), headache impact (HIT-6), and health-related quality of life (MSQ) **FC outcome: RA vs. SA:** ↑Connectivity in the right AMYG/MCC-left MTG and right MCC-right STG **Correlation:** A negative correlation was established between the clinical effects and increased rsFC in the right AMYG/MCC-left MTG |
| Chen 2022, China | **Clinical outcome:** ↓The frequency of migraine attacks, the VAS score, SAS scores, and SDS scores ↑MSQ scores **dALFF outcome: Pre- vs. HCs:** ↓dALFF variability in the RVM, superior lobe of left cerebellum (Cerebellum_Crus1_L), right inferior frontal gyrus, triangular part (IFGtriang. R), right median cingulate, paracingulate gyri (DCG.R), right precuneus (PCUN.R), left Inferior parietal, supramarginal, and angular gyri (IPL.L) ↑dALFF variability in the left Inferior occipital gyrus (IOG.L) **After the ﬁrst and 12th acupuncture treatment sessions vs. the baseline:** ↑dALFF variability of RVM, Cerebellum_Crus1_L, and PCUN.R **DEC outcome:** ↑DEC variability in RVM outflow to the right MFG, left insula, right precentral gyrus, and right supramarginal gyrus, and gradually enhanced DEC variability from the right fusiform gyrus inflow to RVM ↑DEC variability was found from Cerebellum_Crus1_L outflow to the left middle occipital gyrus and the left precentral gyrus, from PCUN.R outflow to the right thalamus **Correlation:** The dynamic Granger causality analysis (GCA) coefficients of this DEC variability were negatively correlated with the frequency of migraine attacks and VAS scores after 12th acupuncture sessions |
| Wallasch 2012, Germany | **Clinical outcome:** ↓Headache frequency and intensity **fTCD outcome:** ↓CVR ( Cerebral Valsalva ratio) results for right/left middle cerebral artery measurements |
| He 2023, China | **Clinical outcome:** ↓VAS, PSQI, and MSQ score **ALFF/ReHo outcome: Post- VS. pre-treatment (AG):** ↑The ReHo and ALFF values in brain regions including thalamus, MFG, cerebellum, and precuneus ↓The ReHo and ALFF values in brain regions involved in inferior frontal gyrus, insula, cerebellum, IPL and CG  The ALFF value decreased in the fusiform gyrus but increased in both the lingual gyrus and STG after acupuncture therapy |
| Quan 2024, China | **Clinical outcome (responders):** ↓Headache frequency, NRS (numerical rating scale), and anxiety / depression levels ↑MSQ scores **dFNC outcome:** Improve the thalamocortical abnormality in MWoA patients and help dFNC coupling restore to a pattern closer to that of HCs **Correlation:** The changes in clinical measurements showed a significant association with the changes in dFNC brain states and the changes in thalamic FC variability The changes in thalamic FC variability showed a significant correlation with the changes in dFNC states |
| Yang 2024, China | **Clinical outcome:** ↓The MIDAS (Migraine Disability Assessment Questionnaire) and SF-MPQ (Short-Form McGill Pain Questionnaire) scores **ALFF/DC outcome: Pre- vs. HCs:** ↑ALFF value in the left fusiform gyrus ↓ALFF values in the right angular gyrus, left middle occipital gyrus, bilateral PFC and left IPL ↑DC (degree centrality) values in the bilateral fusiform gyrus, bilateral ITG and right MTG ↓DC values in the right angular gyrus, right superior marginal gyrus, right IPL, right middle occipital gyrus, right SFG, right MFG, right anterior central gyrus, and the right SMA **Post- VS. pre-treatment:** ↓ALFF/DC values of the right ITG, right fusiform gyrus and right MTG **Correlation:** ALFF values in the left middle occipital area were positively correlated with the pain degree at time point 1 (TP1) in Group1 The DC values of the right ITG were positively correlated with the pain degree at TP1 in Group 1 |
| Qin 2019, China | **Clinical outcome:** ↓VAS **ReHo outcome:** **Post- VS. pre-treatment (AG):** ↑ReHo values in the anterior CG, anterior central gyrus, superior orbital frontal gyrus, insula, inferior lobule, let anterior CG, ventral lateral nucleus and ventral posteromedial nucleus of the thalamus, pontine nucleus, cerebellar tonsils and orbital frontal inferior gyrus of the brain ↓ReHo values in the right brain bridge, central posterior gyrus,posterior cingulate gyrus, left central anterior gyrus, posterolateral nucleus of thalamus, and hippocampus **Post- VS. pre-treatment (SA group):** ↑ReHo values in the right tongue gyrus, the left anterior lobe, the anterior cingulate gyrus and the lower occipital gyrus ↓ReHo value in the left ventral posterolateral nucleus of the thalamus |
| Liu 2022, China | **Clinical outcome:** ↓Headache days, VAS score, the total score of headache symptoms, SAS score and SDS score ↑The scores of the restrictive, preventive, and emotional functional domains of the MSQ **FC outcome:** **Pre-acu VS. HC:** ↓FC between PAG and the right cerebellum Ⅷ **Post- VS. pre-treatment:** ↑FC of PAG with the bilateral cerebellum Ⅷ and the left precuneus **Correlation: Pre-treatment:** FC intensity of PAG and the right cerebellum Ⅷ was negatively correlated with VAS score **Post-treatment:** FC intensity of PAG and the left precuneus was positively correlated with the improvement in headache days |
| Fu 2024, China | **Clinical outcome:** ↓Days of headache episodes, number of episodes, and Level of headache (VAS score) **MRS outcome:** **Pre- VS. HC:** ↓NAA/Cr and NAA/Cho (Choline compounds) of left thalamus **Post- VS. pre-treatment:** ↑NAA/Cr and NAA/Cho of left thalamus and NAA/Cr of right thalamus **Post- VS. HC:** There was no statistical difference between the control group and the control group **Correlation:** The NAA/Cr change of left thalamic before and after treatment was negatively correlated with the change of headache severity score (VAS score) |
| Zheng 2013, China | **Clinical outcome:** ↓Headache days, headache counts, VAS scores **TCD outcome:** **Post- VS. pre-treatment (Shaoyang points group vs. non-acupoints group):** The mean flow of Left middle cerebral artery (L-MCA) is significantly lower in Shaoyang points group than non-acupoints group |
| Liang 2016, China | **Clinical outcome:** ↓VAS **H - MRS outcome:**  **Post- VS. pre-treatment (Shaoyang points group vs. non-acupoints group):** NAA／Cr ration at the PAG of the treatment group was higher than the control group’s |
| Chen 2009, China | **TCD outcome:** Cerebral blood flow velocity was significantly improved in the group of Shaoyang points group, whereas no significant changes were seen in the non-acupuncture points group |
| Lin 2013, China | **Clinical outcome:** ↓The times of attack, lasting time, accompanying symptoms, and the intensity of attack **TCD outcome:** **Post- VS. pre-treatment (Shaoyang points group):** ↓Both sides peak systolic velocity of contraction period of cerebralarteries including the Vp (Vmax, peak velocity, maximum velocity) of MCA, anterior cerebral artery (ACA), PCA, vertebral artery (VA), basilar artery (BA) **Post- VS. pre-treatment (Shaoyang points group vs. non-acupoints group):** Except for the both sides peak systolic velocity of ACA, RMCA and LVA, the two sides peak systolic velocity of PCA, RVA and BA had significant difference between the two groups after treatment |
| Zhang 2020, China | **Clinical outcome:** ↓Headache attacks, VAS, and headache intensity **fMRI outcome:** **Pre-treatment MM vs. HC:** Compared with healthy subjects, the functional connectivity of the precuneus with MFG, precuneus with caudate nucleus were significantly lower in menstrual migraine patients  **Post- VS. pre-treatment:** ↑The functional connection of the precuneus with MFG, precuneus with caudate nucleus **Correlation:** The functional connectivity change of precuneus with MFG, precuneus with caudate nucleus after acupuncture was negatively correlated with VAS |
| Xu 2023, China | **Clinical outcome:** ↓VAS **fMRI outcome:** **Pre- vs. HC:** ↓DC in the left SMA, limbic lobe, medial aspect of the frontal gyrus, and the middle of the left CG The clustering coefficients and local efficiencies of the MWoA were significantly decreased, with a decreasing trend in the shortest path, the small-world index, and the global efficiency **Post- VS. pre-treatment:** ↑The clustering coefficient, local efficiency, small world index, and global efficiency |
| Yu 2023, China | **DT-MRI outcome:** **Pre- VS. HC:** The FA (fractional anisotropy) values of genu, body and splenium of corpus callosum in the observation group were significantly lower than those in the control group **Post- VS. pre-treatment:** The FA values of the knee, body and pressure parts of the corpus callosum were significantly higher in the effective patients than in the ineffective patients |
| Zhang 2022, China | **Clinical outcome:** ↓number of days of migraine attack, VAS, HIT-6, MIDAS, BAI (Beck Anxiety Inventory), BDI (Beck Depression Inventory) **rs-MRI outcome:** **Pre- VS. HC:** ↓voxel-mirrored homotopic connectivity (VMHC) in the bilateral cerebellum_Ⅷ, cuneus and postcentral gyrus **Post- VS. pre-treatment:**  ↑Weakened VMHC of the bilateral cuneus **Correlation:** The change of VMHC in the bilateral cuneus was positively correlated with change of SAS scores |
| Xu 2023, China | **Clinical outcome:** ↓Headache time and VAS FA outcome: Pre- VS. HC: ↓FA values in some nodes of the right corticospinal tract and right superior longitudinal fasciculus Post- VS. pre-treatment:  ↑FA values in some nodes of the right corticospinal tract, right superior longitudinal fasciculus, and left CG |

**Abbreviations:** VA1, VA2, VA3: verum acupuncture groups 1, 2, 3; ICA, independent component analysis; MRS, magnetic resonance spectroscopy; CM, chronic migraine; zALFF, z-transformed amplitude of low-frequency fluctuation; TA, true acupuncture; SA, sham acupuncture; RA, real acupuncture; DEC, dynamic effective connectivity; f-TCD, functional transcranial Doppler sonography; dFNC, Dynamic functional network connectivity; MM, menstrual migraine; DT-MRI, diffusion tension-magnetic resonance imaging; rs-fMRI, resting-state functional magnetic resonance imaging.
